# Determination of the pH dependence, substrate specificity, and turnovers of alternative substrates for human ornithine aminotransferase

**DOI:** 10.1016/j.jbc.2022.101969

**Published:** 2022-04-20

**Authors:** Arseniy Butrin, Anastassiya Butrin, Zdzislaw Wawrzak, Graham R. Moran, Dali Liu

**Affiliations:** 1 Department of Chemistry and Biochemistry, Loyola University Chicago, Chicago, Illinois, USA; 2 Synchrotron Research Center, Life Sciences Collaborative Access Team, Northwestern University, Argonne, Illinois, USA

**Keywords:** human ornithine aminotransferase, mechanism-based inactivators, hepatocellular carcinoma, pH dependence, substrate specificity, alternative substrates, enzyme kinetics, pyridoxal phosphate, GABA, AVA, 5-aminovaleric acid, CCD, charge-coupled device, DABA, l-2,4-diaminobutyric acid, 5-FMeOrn, 5-fluoromethylornithine, GABA, γ-aminobutyric acid, HCC, hepatocellular carcinoma, *h*OAT, human ornithine aminotransferase, α-KG, α-ketoglutarate, l-GSA, l-glutamate-γ-semialdehyde, l-Orn, l-ornithine, MAT, MES, acetic acid, Tris, and NaCl buffer, MBI, mechanism-based inactivator, P5C, Δ1-pyrroline-5-carboxylate, PDB, Protein Data Bank, pHi, intracellular pH, PLP, pyridoxal-5′-phosphate, PMP, pyridoxamine phosphate

## Abstract

Hepatocellular carcinoma (HCC) is the most common primary cancer of the liver and occurs predominantly in patients with underlying chronic liver diseases. Over the past decade, human ornithine aminotransferase (*h*OAT), which is an enzyme that catalyzes the metabolic conversion of ornithine into an intermediate for proline or glutamate synthesis, has been found to be overexpressed in HCC cells. *h*OAT has since emerged as a promising target for novel anticancer therapies, especially for the ongoing rational design effort to discover mechanism-based inactivators (MBIs). Despite the significance of *h*OAT in human metabolism and its clinical potential as a drug target against HCC, there are significant knowledge deficits with regard to its catalytic mechanism and structural characteristics. Ongoing MBI design efforts require in-depth knowledge of the enzyme active site, in particular, p*K*a values of potential nucleophiles and residues necessary for the molecular recognition of ligands. Here, we conducted a study detailing the fundamental active-site properties of *h*OAT using stopped-flow spectrophotometry and X-ray crystallography. Our results quantitatively revealed the pH dependence of the multistep reaction mechanism and illuminated the roles of ornithine α-amino and δ-amino groups in substrate recognition and in facilitating catalytic turnover. These findings provided insights of the catalytic mechanism that could benefit the rational design of MBIs against *h*OAT. In addition, substrate recognition and turnover of several fragment-sized alternative substrates of *h*OATs, which could serve as structural templates for MBI design, were also elucidated.

Human ornithine aminotransferase (*h*OAT) (1) is an enzyme that catalyzes the transfer of the δ-amino group from l-ornithine (l-Orn) to α-ketoglutarate (α-KG). As a pyridoxal-5′-phosphate (PLP)–dependent transaminase, *h*OAT has a “Bi−Bi, Ping–Pong” kinetic mechanism. In the first half-reaction, PLP and l-Orn are converted to pyridoxamine phosphate (PMP) and l-glutamate-γ-semialdehyde (l-GSA). l-GSA is then prone to cyclize to Δ1-pyrroline-5-carboxylate (P5C). In the second half-reaction, *h*OAT catalyzes the transfer of the amino group of PMP to an α-keto acid, preferentially α-KG, forming glutamate and regenerating the PLP form of the cofactor (Fig. 1) .

**Figure 1:**
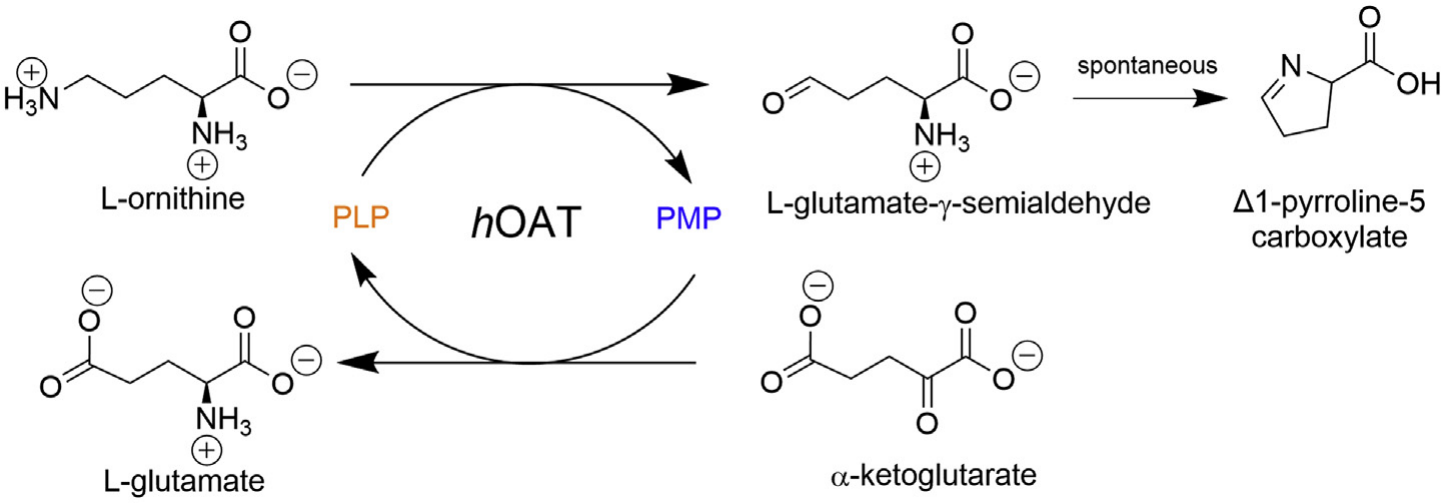
**Summarized half-reactions of *h*OAT.***h*OAT, human ornithine aminotransferase.

In mammals, OAT makes a crucial contribution to multiple metabolic pathways, including glutamine metabolism, proline and arginine biosynthesis, and the urea cycle. In addition, it was found that OAT plays a crucial role in the early development of neonates (2) for the reason that mammalian milk is a poor source of arginine, and thus, it must be synthesized from citrulline whose concentration is regulated by OAT in the small intestine (3). The conversion of significant amounts of proline from maternal milk into arginine has been confirmed for human, pig, and mouse neonates (2, 3, 4, 5). Deficiency and inhibition of OAT in humans was found to cause gyrate atrophy (6) and hyperornithinemia (7).

While some slow-onset diseases are associated with insufficient *h*OAT activity, studies have also shown that overexpression of *h*OAT supports the development and proliferation of cancer cells (8, 9). Hepatocellular carcinoma (HCC) is the most common form of primary liver cancer, accounting for 90% of all cases of liver cancer in the United States (10, 11, 12, 13). If diagnosed early, HCC can be treated effectively with surgery. In the latter stages, additional treatment is required including radiotherapy and chemotherapy (14, 15, 16, 17). In reality, HCC is typically diagnosed in advanced stages when proliferated tumors are resistant to both radiotherapy and chemotherapy. Recently published works have shown that *h*OAT and other glutaminogenic enzymes were found to be overexpressed in HCC cells because of abnormal oncogenic Wnt/β-catenin signaling (8, 9). Thus, *h*OAT has been identified as a potential drug target for novel anticancer therapy against HCC. *In vivo* inhibition of *h*OAT in the HCC mouse models have demonstrated encouraging results for a potential antitumor effect by mechanism-based OAT inactivators (18).

Despite the significance of *h*OAT in human metabolism and its established contribution to HCC development, many fundamental properties of this enzyme remain unknown. Like several other aminotransferases, OAT is a promiscuous enzyme, and a number of its alternative substrates have been identified experimentally (19). But detailed mechanistic and structural analysis of the enzyme's interaction with alternative substrates is currently lacking. Herein, fundamental mechanistic questions of *h*OAT are addressed. A pH profile for the rate-limiting chemical step(s) was generated, and key kinetic p*K*a values were determined. Transient-state kinetic experiments on l-Orn and three other smaller alternative substrates have been performed (Fig. 2), and crystal soaking experiments were employed to trap intermediate states for these ligands. The results reveal the importance of substrate α-amino and δ-amino groups in substrate recognition and catalysis. The data obtained serve as a structural and mechanistic basis for the development of new *h*OAT inhibitors and/or mechanism-based inactivators that mimic the fragment-sized alternative substrates, such as γ-aminobutyric acid (GABA) or 5-aminovaleric acid (AVA).

**Figure 2:**
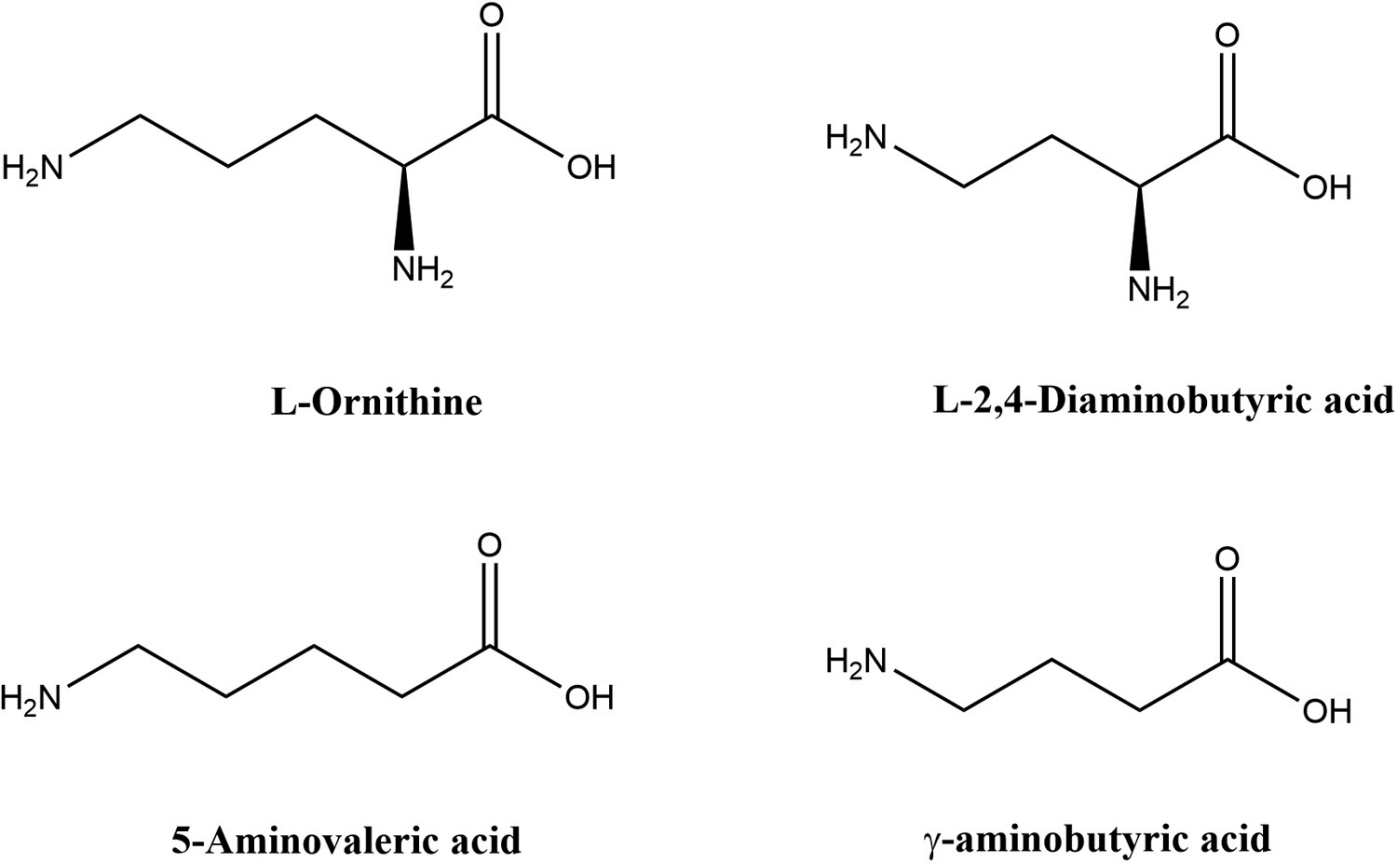
**Chemical structures of****l****-ornithine and three alternative substrates:****l****-2,4-diaminobutyric acid (DABA), 5-aminovaleric acid (AVA), and γ-aminobutyric acid (GABA)**.

## Results

Among the unanswered questions pertaining to the structural and mechanistic properties of *h*OAT, one of them addressed in this work is the determination of the macroscopic p*K*a values that govern the enzyme’s half-reactions. It has been previously shown that the kinetics of each half-reaction of rat liver OAT is influenced by pH; however, those experiments were largely qualitative (20) in that working pH ranges of the enzyme were identified without measurement of the macroscopic p*K*a for either half-reaction. In the current work, the pH profile of *h*OAT was observed for the forward half-reaction using l-Orn and for the reverse half-reaction using α-KG using stopped-flow spectroscopy. To augment the kinetic experiments, holo-*h*OAT was crystallized at a pH of 6.0. The protein crystal diffracted to a resolution of 2.1Å and revealed notable changes in the orientation of specific active-site residues. To investigate substrate specificity of *h*OAT, transient-state kinetic experiments were conducted with three analogs of l-Orn: AVA, GABA, and l-2,4-diaminobutyric acid (DABA) (Fig. 2). All analogs exhibited relatively slow catalytic rates; therefore, a series of *h*OAT crystal soaking experiments were conducted in an attempt to capture structures of their reaction intermediates. The soaking experiments resulted in a structure of GABA covalently attached to the PLP as well as the structure of AVA linked both to PLP and the catalytic Lys292.

### pH dependence of the half-reactions of *h*OAT

**Figure 3:**
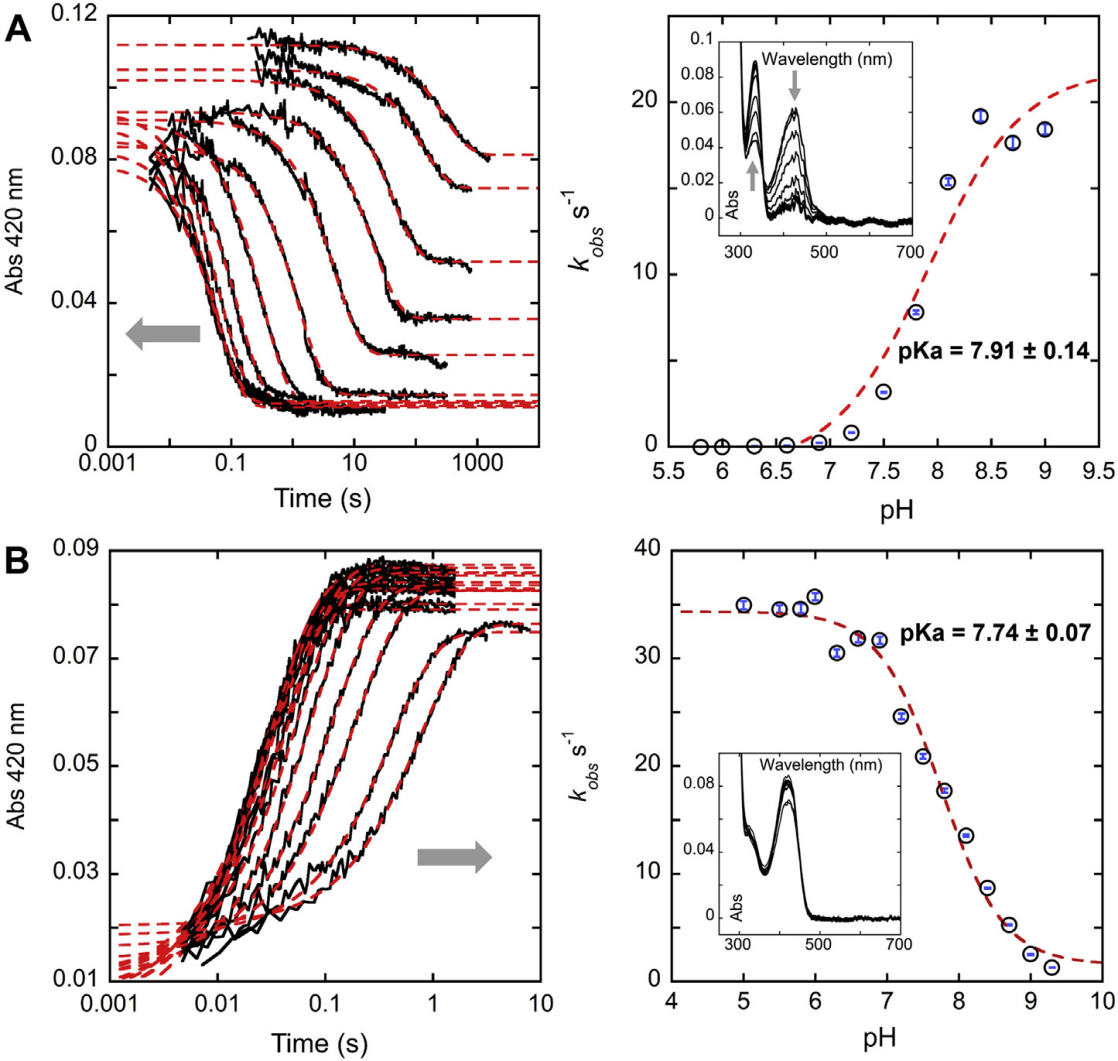
***h*OAT pH dependence plots for the first half-reaction.** With l-Orn (*A*) and the second half-reaction with α-KG (*B*). Each kinetic trace was fit to Equation 1 to determine observed rate constants (*k*_obs_). The pH dependence of the *k*_obs_ values was fit to Equation 2. In each plot to the *left*, the *gray arrow* indicates the trend toward higher pH values. The observed rate was measured at pHs 5.8, 6.0, 6.0, 6.3, 6.6, 6.9, 7.2, 7.5, 7.8, 8.1, 8.4, 8.7, and 9.0. The *markers* are shown as *hollow black circles* overlaying *blue error bars*, the magnitudes of which are derived from the fit to Equation 2. Spectra extracted at equilibrium are shown as the *insets* and represent the balance of aldimine and PMP forms of the enzyme. *h*OAT, human ornithine aminotransferase; α-KG, α-ketoglutarate; l-Orn, l-ornithine; PMP, pyridoxamine phosphate.

PLP-dependent enzymes exhibit prominent signature spectrophotometric absorption characteristics that facilitate observation of their reactions. By monitoring the spectrum of the cofactor in turnover, explicit details of the reaction mechanism can be elucidated (21, 22). For pH-dependent kinetic studies of *h*OAT, transient-state pH-jump experiments were conducted using the stopped-flow instrument. These data are shown in Figure 3.

Both reactions were observed under pseudo–first-order conditions using charge-coupled device (CCD) detection from 250 to 800 nm. In both cases, the data are described by the fit to a single exponential and thus report only interconversion between the external aldimine and PMP. From the pH profiles, it was determined that the first half-reaction with l-Orn approaches the maximum rate at ≥pH 9.5. The kinetic p*K*a was calculated to be 7.91 ± 0.14. For the second half-reaction with α-KG, the maximum reaction rate was observed at pH ≤6.0, and it decreased with higher pH. The kinetic p*K*a was determined as 7.74 ± 0.07. The observed small difference in the p*K*a values may indicate titration of the same group acting in the rate-limiting catalytic step of both the first and second half-reactions, such as the tethering Lys292. Although the solution p*K*a value for the ε-amino group of lysine is 10.53 (23), the p*K*a of Lys292 may be shifted to a lower value in the active site of *h*OAT as has been observed for aspartate aminotransferase (24).

Spectra extracted at equilibrium represent the balance of aldimine and PMP forms of the enzyme (Fig. 3, *insets*). These spectra indicate that the first half-reaction is biased forward to the PMP state only at high pH values and that the product of the first half-reaction, l-GSA, is a substrate for the second half-reaction, dictating that ring closure to P5C does not occur on the surface of the enzyme. Conversely, the second half-reaction with α-KG as a substrate is strongly biased toward aldimine and l-glutamate formation at all pHs.

### Crystal structure of holo-*h*OAT at pH 6.0

**Figure 4:**
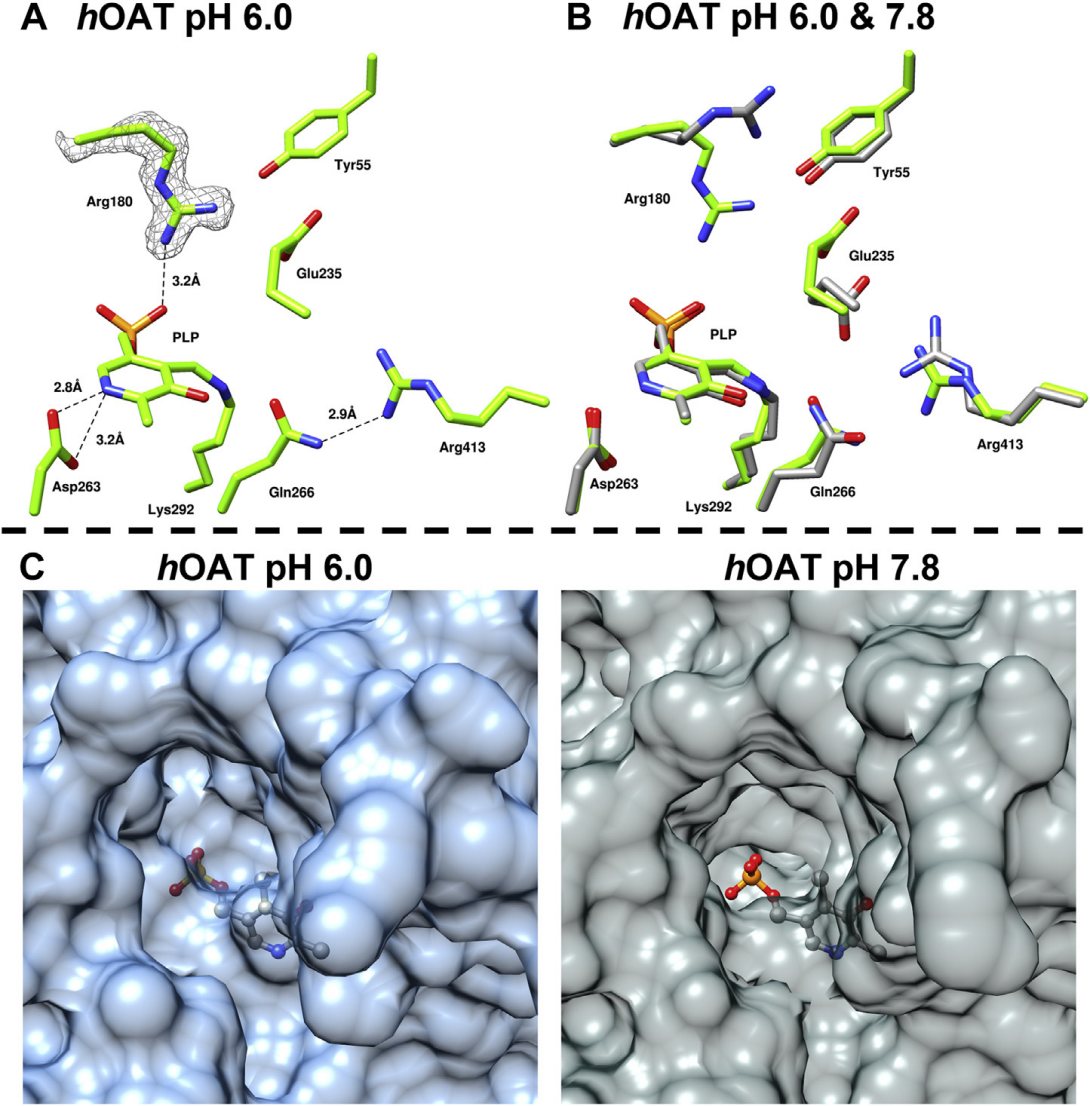
**Comparison of the active-site structure of *h*OAT at pH 6.0 and 7.8.***A*, active site of *h*OAT crystallized at pH 6.0 (Protein Data Bank ID: 7T9Z). The electron density (simulated annealing composite 2*F*_o_–*F*_c_ map at 1.1 σ) is shown as a *gray contour* around Arg180. *B*, overlayed structures of *h*OAT at pH 6.0 (*green*) and pH 7.8 (*gray*). *C*, comparison of *h*OAT homodimer surface within active-site cavity at pH 6.0 (*left*) and pH 7.8 (*right*). Active-site residues and PLP are shown as *sticks*. *Dashed black lines* indicate hydrogen bonds. *h*OAT, human ornithine aminotransferase; PLP, pyridoxal-5′-phosphate.

The observed rate decrease in the first half-reaction of *h*OAT with l-Orn at low pH in stopped-flow experiments indicates that deprotonation of one or more active-site groups enhances the rate of the reaction. To structurally assess the influence of low pH values, an X-ray crystal structure of *h*OAT at pH 6.0 was obtained with a resolution of 2.1 Å. This structure was solved using molecular replacement (search model Protein Data Bank [PDB] ID: 1OAT (25)). After model building and refinements, the final model presents three monomers in an asymmetric unit of the C 1 2 1 space group (Table S1). The active site of the structure of *h*OAT internal aldimine state at pH 6.0 and 7.8 is depicted in Figure 4.

Although no major structural changes were detected in the overall quaternary and tertiary structure of *h*OAT at pH 6.0, some notable deviations were observed in local conformations of the active-site residues. Acidic conditions altered the position of the Arg180 side chain, a residue responsible for initial substrate recognition and binding (25, 26). In addition, the side chain of Glu235 exhibited relatively weak electron density reflecting increased mobility of this residue at lower pHs. In the refined low pH model, the side chain of Glu235 faces away from Arg413 (Fig. S2). The Arg413–Glu235 salt bridge is proposed to act as a molecular switch that prevents a nonproductive interaction between l-Orn and Arg413 in the first half-reaction (27). In the pH 6.0 structure, Arg180 moved toward the center of the active site occupying the space where the substrate would be localized. As shown in Fig. S3, side chains of Arg180 and Glu235 that regulate the width of the active-site pocket define the size of the channel through which the substrate ligand passes. At pH 7.8, the distance between the two side chains is 7.8 Å, but it reduces to 5.7 Å at pH 6.0. This results in the constriction of the active-site pocket as shown in protein surface comparison in Figure 4*C*. Such conformational alterations for Arg180 and Glu235 could directly alter binding affinity for substrates and/or contribute to the observed decrease in catalytic rate (Fig. 3). Moreover, with the loss of interaction between Arg413 and Glu235, there is the potential for the carboxyl group of l-Orn to form a charge-pairing interaction with Arg413 resulting in nonproductive binding. The observed conformational changes for Arg180 and Glu235–Arg413 could thus account for the decreased reaction rate at low pH in the first half-reaction or could be additive with changes in the protonation state of other active-site residues and/or PLP that do not induce conformational alterations and so are not observed crystallographically.

### Transient-state measurements of *h*OAT reaction with alternative substrates

**Figure 5:**
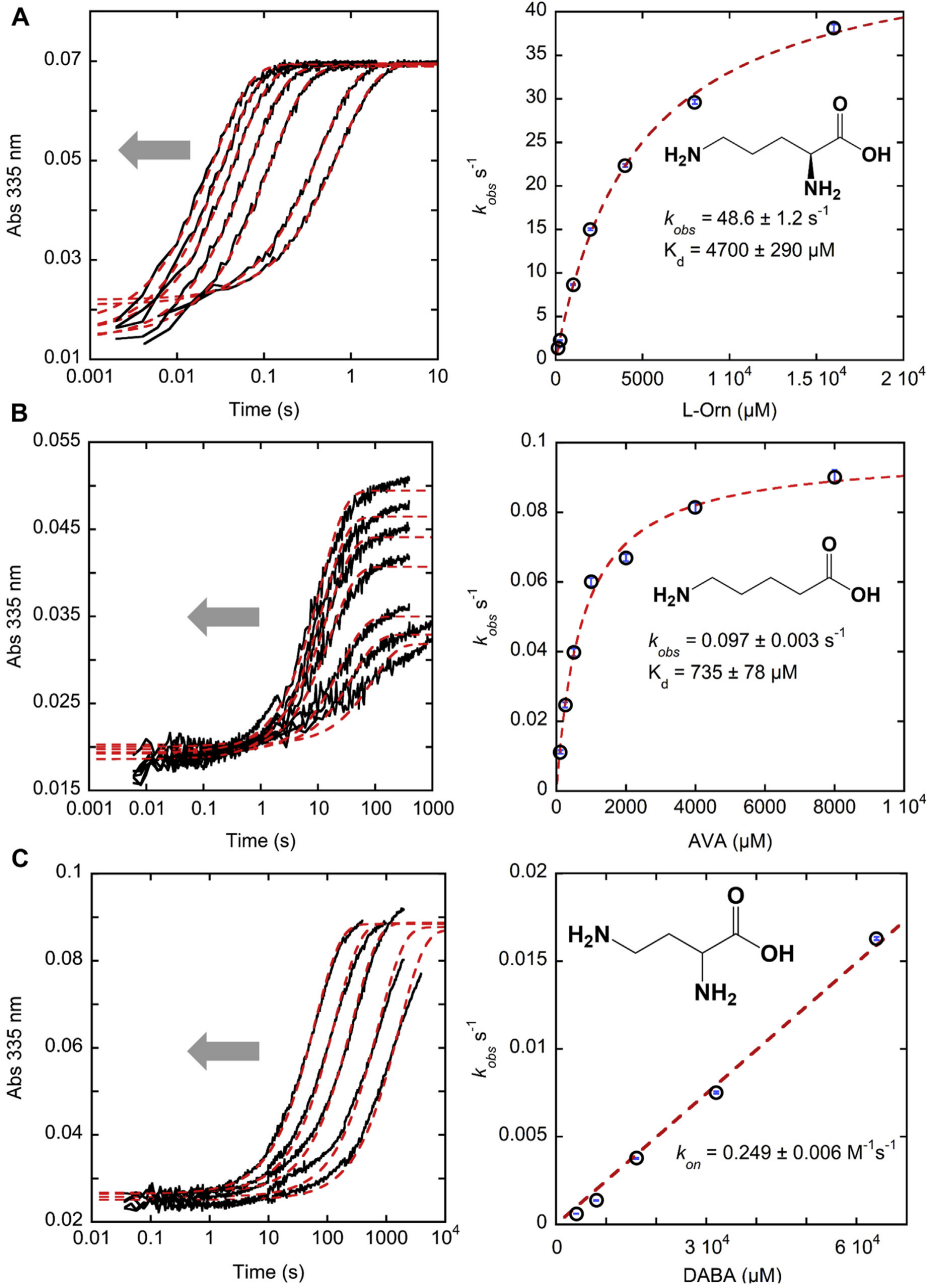
**Transient-state absorption changes observed at 335 nm for *h*OAT.***h*OAT reacting with l-Orn (*A*), AVA (*B*), and DABA (*C*). *h*OAT concentration for the reaction with l-Orn and AVA was 9 μM. For the studies with DABA, the enzyme concentration was 10.4 μM. The observed rate constants were plotted against substrate concentrations and fit to hyperbolic function (l-Orn and AVA; Equation 3) or linear function (DABA; Equation 4). The direction of the *gray arrow* in the plots to the *left* indicates the trend of substrate’s concentration change toward higher values. The markers are shown as *hollow black circles* overlaying the *blue relative error bars*. AVA, 5-aminovaleric acid; DABA, l-2,4-diaminobutyric acid; *h*OAT, human ornithine aminotransferase; l-Orn, l-ornithine.

Despite the fact that *h*OAT is mainly characterized as a δ-aminotransferase, several recent publications have shown that the enzyme is capable of reacting with cyclized ligands in which the amino group was located in γ position (28, 29, 30, 31). Moreover, it was demonstrated that l-glutamate with a single α-amino group could also serve as a substrate in the first half-reaction (32). Other studies of *h*OAT have examined variants at Tyr55 and Arg180 and confirmed their role in anchoring l-Orn through noncovalent interactions with carboxylate and α-amino group, respectively (33, 34). However, how the active-site recognition of δ-amino and α-amino groups of l-Orn as well as how the substrate size contributes to both binding and catalysis have not been investigated in detail. The mechanistic and structural determinants of the substrate preference of *h*OAT for l-Orn are not clear, as aminotransferases are commonly known for their catalytic promiscuity (35, 36). To explore the determinants of substrate selectivity, three substrate analogs of l-Orn were selected and reacted with *h*OAT. The results of transient-state kinetic experiments were compared with that of l-Orn. Each compound was titrated against *h*OAT, and the absorption changes associated with the reaction were observed. The results of transient-state absorption changes at 335 nm, which report the formation of PMP produced in the first half-reaction for all tested substrates, are shown in Figures 5 and S4. The CCD spectra for the highest concentrations of the substrates are shown in separate 3D plots (Figs. S5–S8).

For each ligand including l-Orn, it was evident that an early step of the reaction was rate limiting; hence, no intermediates after the formation of the external aldimine (that forms in the dead time) were observed. This early rate-limiting step is assigned to the conversion of external aldimine to the quinonoid state. Comparison of the observed spectra with the initial spectra after the addition of the AVA, GABA, or DABA revealed a small red shift from ∼420 nm to ∼425 nm. This happened within a dead time of the instrument and was assigned as the conversion of internal to external aldimine (Fig. S9). The observed small red shift is a unique signal for the external aldimine formation for the alternative substrates. For l-Orn, the conversion of internal to external aldimine was less apparent than those of other ligands presumably because the structure of the external aldimine complex closely resembles the internal aldimine tethered by the catalytic Lys292. Nevertheless, the existence of external aldimine in the reaction with l-Orn was validated through the following pH-dependent phenomenon. The absorption spectra of 9 μM holo-*h*OAT were measured and compared at different pH values. No observable shift of the internal aldimine peak was detected (Fig. S10). Then, initial absorption spectra of the reaction between 36 μM *h*OAT and 2 mM l-Orn at different pH values were compared (Fig. S10). It transpired that the peak observed at ∼420 nm at pH 9.0 is progressively shifted to ∼425 nm at lower pH values. This transition was assigned to the different protonation states of the external aldimine, where the absorption peak of the protonated form is red shifted compared with the deprotonated form.

The reaction traces with l-Orn, AVA, and DABA were fit to a single exponential (Equation 2) to obtain sets of observed rate constants. For each substrate, the observed rates were plotted against substrate concentrations and fit to hyperbolic function (l-Orn and AVA, Equation 3) or linear function (DABA, Equation 4). The kinetic data are summarized in Table 1.

**Table 1:** Summary of kinetic parameters for *h*OAT using various substrates

Substrate	Limiting rate constant (*k*_lim_)	Dissociation constant (*K*_*d*_)	*k*_lim_/*K*_*d*_
l-Orn	48.6 ± 1.2 s^−1^	4700 ± 290 μM	10,340 ± 687 M^−1^ s^−1^
AVA	0.097 ± 0.003 s^−1^	735 ± 78 μM	131.9 ± 14.6 M^−1^ s^−1^
DABA	ND	ND	0.249 ± 0.006 M^−1^ s^−1^
GABA	ND	110 ± 15 μM	ND

Abbreviation: ND, not determined.

The native substrate l-Orn possesses the highest observed limiting rate constant and highest pseudo–second-order rate constant *k*_lim_/*K*_*d*_, which is a measure of substrate specificity in the first half-reaction. AVA exhibited higher binding affinity to *h*OAT, ∼6.4-fold that observed for l-Orn but reacted ∼500-fold more slowly. For DABA, the dependence of the observed rate on substrate concentration expressed linear character from 4 to 64 mM, indicating that the reaction of *h*OAT with DABA is weak and/or has a diminished tendency to form the external aldimine state. The slope of the line represents the second-order rate constant *k*_lim_/*K*_*d*_ for DABA. *h*OAT therefore has ∼41,500-fold less specificity for DABA than for l-Orn and ∼530 times less for AVA.

Among the four substrates studied, GABA expressed the highest binding affinity toward *h*OAT. However, in reaction with GABA, the full decay of external aldimine was never achieved even when the enzyme was fully saturated possibly because of a bias in terms of binding and rate for the second half-reaction returning the enzyme to the external aldimine state. The binding of GABA was confirmed spectrophotometrically by the rapid and small red shift of absorbance peak from ∼420 nm (internal aldimine) to ∼425 nm (external aldimine) within the dead time of the stopped-flow instrument. The subsequent decay of external aldimine and formation of PMP went slowly and was completed in 1000 s (Fig. S4). According to the observed change in the amplitude of external aldimine at 425 nm, only a fraction of the enzyme accumulated as the PMP state at equilibrium (Fig. S4). The *K*_*d*_ of GABA was measured separately and was estimated to be 110 ± 15 μM, ∼40-fold lower than that for l-Orn and ∼6 times lower than that for AVA.

### Crystal structures of *h*OAT soaked with GABA and AVA

**Figure 6:**
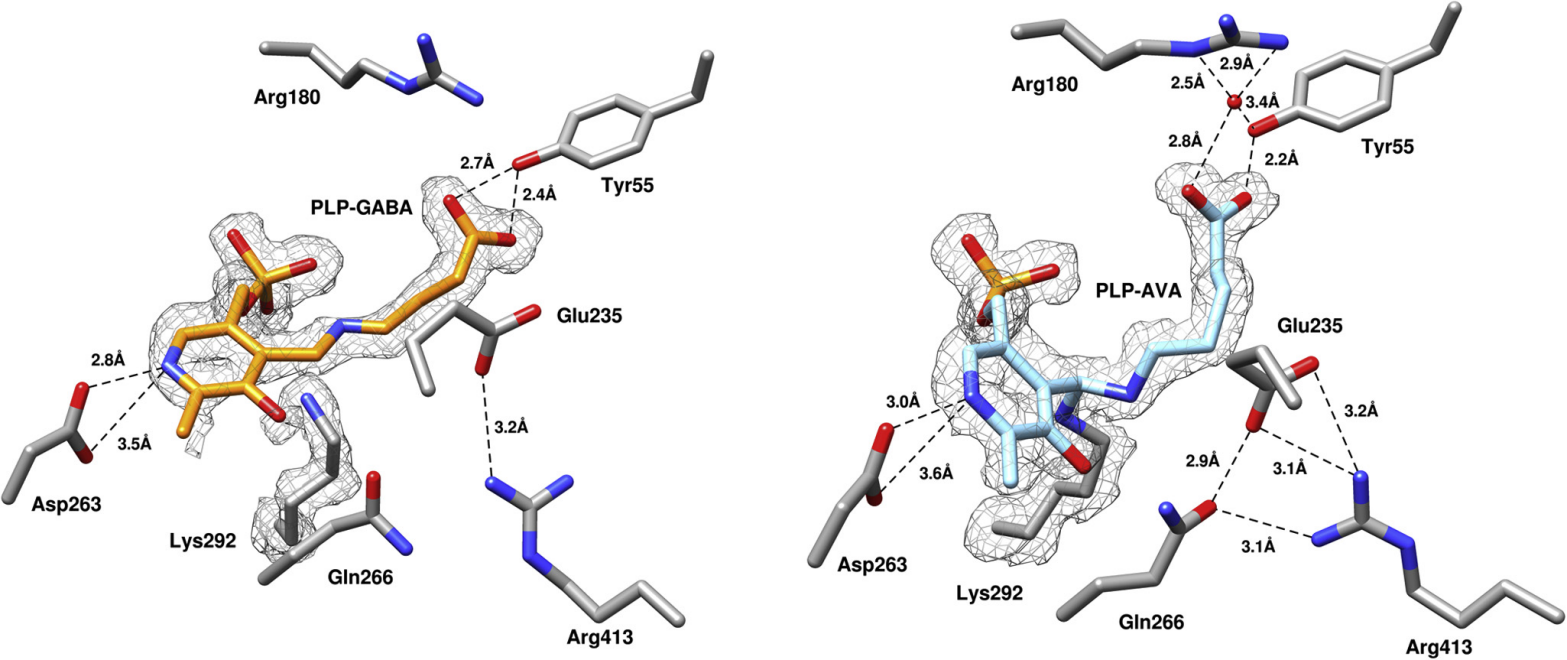
**Polder map (*F***_**o**_**–*F***_**c**_**, at 3.0 σ) of the *h*OAT structure soaked with GABA (*left*, Protein Data Bank ID:****7TA1****) and AVA (*right*, PDB ID:****7TA0****).** In both structures, the reaction intermediates of GABA and AVA with *h*OAT are shown within its active site. Based on the observed electron density and transient kinetic results, an external aldimine intermediate state was built for the reaction with GABA, whereas gem–diamine intermediate was built for the reaction with AVA. *Dashed black lines* indicate hydrogen bonds or charge interactions. Electron density maps are shown as *gray mesh* around the ligands and catalytic lysine. AVA, 5-aminovaleric acid; GABA, γ-aminobutyric acid; *h*OAT, human ornithine aminotransferase.

The transient-state kinetics of *h*OAT using alternative substrates suggest possible diversity in their binding modes and chemical mechanisms. Much like observations made for l-Orn, the decay rate of external aldimine species is the rate-limiting process in the first half-reaction for all alternate substrates. Soaking experiments of holo-*h*OAT crystals were prepared in an attempt to trap the reaction intermediates for all three compounds under the assumption that the catalysis may occur slowly in crystallo. For each ligand, the crystals were soaked for various time intervals from 3 min to an hour for AVA and DABA and from 50 min to 2.5 h for GABA. The crystals soaked with GABA for 1 h and 30 min and the crystals soaked with AVA for 30 min diffracted to similar resolutions of ∼2.2 Å. DABA crystals had poor diffraction and were omitted from further structural analysis. Complex structures were solved with molecular replacement (search model PDB ID: 1OAT (25)). *h*OAT–GABA crystal structure was processed in the P 3_1_ 2 1 space group, whereas that of *h*OAT–AVA was processed as P 3_2_ 2 1. The active sites for structures of *h*OAT soaked with GABA and AVA are shown in Figure 6.

In both soaking experiments, reaction intermediates were trapped. For GABA, an intermediate formed that exhibited a covalent linkage with PLP and was detached from the catalytic Lys292, a structure consistent with the proposed external aldimine intermediate. The carboxylate group of the ligand formed two hydrogen bonds with Tyr55 instead of interacting with Arg180, which is proposed to interact with the carboxylate of l-Orn (37). No other interactions were observed between the ligand and the active-site residues. For two monomer copies in an asymmetric unit, the Glu235–Arg413 salt bridge that is a key feature in the holo-*h*OAT was found partially disrupted: the side chain of Arg413 moved away from Glu235 and Gln266. In other monomer copies, the Glu235–Arg413 salt bridge remained intact. Previous kinetic experiments with GABA carried out by Markova *et al.* (33) showed that *h*OAT has the capacity to process GABA into succinic semialdehyde despite a low rate constant (*k*_*lim*_ = 0.006 ± 0.004 s^−1^ at 25 °C). Considering the exceedingly slow reaction rate, relatively short soaking time, and good fit of the model into the observed electron density, it can be concluded that the species observed in the structure is not a final adduct but rather an external aldimine intermediate.

The structure of *h*OAT crystal soaked with AVA revealed an intermediate that closely resembles the gem–diamine intermediate (Fig. S1). Unlike other intermediates in a typical transamination reaction, the gem–diamine is an early intermediate in which the catalytic lysine, the PLP, and the substrate are covalently joined together, and the observed electron density can be fit convincingly to such an intermediate state (Fig. 6). The carboxylate group of AVA formed a strong hydrogen bond (measured as 2.2 Å) with Tyr55 and indirectly interacted with Arg180 through hydrogen bonds *via* a water molecule, and the Glu235–Arg413 salt bridge remained intact. That this intermediate is observed in the crystal indicates that the lattice greatly modifies the magnitude and relative rates of the chemistry, in that no evidence for a gem–diamine species was observed for the same reaction in solution (Figs. 5 and S6).

The intermediate structures obtained for GABA and AVA reactions provide the evidence for interactions that happen with the substrates at the early reaction stages. These results can be compared with the previously published structure of *h*OAT with its inhibitor 5-fluoromethylornithine (5-FMeOrn), which mimics the productive external aldimine intermediate of *h*OAT with l-Orn (PDB ID: 2OAT). This model has been accepted as the structural doctrine for substrate and external aldimine binding for *h*OAT. The comparison of obtained intermediate structures with 5-FMeOrn–*h*OAT is shown in Figs. S11 and S12. Since both AVA and GABA lack an α-amino group, they are compelled to interact with Tyr55 through their carboxylate groups. No direct interactions with Arg180 were observed for AVA and GABA intermediates although AVA was found to interact with it indirectly through the water molecule. The Glu235–Arg413 salt bridge was found intact both for AVA and 5-FMeOrn structures but not for GABA structure. The Glu235–Arg413 salt bridge is proposed to prevent nonproductive binding of substrate (l-Orn) to Arg413 (37) in the first half-reaction. However, Arg413 is involved in the second half-reaction in which it interacts with dicarboxylic substrates (α-KG) (25). The salt bridge in the GABA structure was found disrupted in two of six monomers in an asymmetric unit. It is possible that the salt bridge could be broken during the first half-reaction to prepare the enzyme for the second half-reaction. Alternatively, an equilibrium state of the protein could have been observed in those copies. In the equilibrium state, the reverse reaction with the product of GABA (succinic semialdehyde) could be taking place in those monomer copies disrupting the Glu235–Arg413 salt bridge. As another option, the observed salt bridge disruption could also be a crystallographic artifact.

The crystal structure of AVA also provided a curious insight on the early steps of the catalysis. The obtained structure of the gem–diamine was compared with structures of the internal aldimine (PDB ID: 1OAT) and external aldimine of the 5-FMeOrn (PDB ID: 2OAT) in Fig. S13. The overlap of these three structures revealed two different orientations of PLP in the active site of *h*OAT. Internal aldimine and gem–diamine were found anchored by the side chain of catalytic Lys292 tilting the pyridine ring of PLP. By contrast, the external aldimine is free from Lys292 and is distanced 3.4 Å from its ε-amino group. Without the structural restraint from Lys292, the pyridine ring of the PLP tilts notably in the direction of Arg180. The angles between the pyridine planes were measured as 29.4° between gem–diamine and external aldimine and 24.3° between internal and external aldimine, respectively. The energy released from the structural restrain could facilitate the formation of external aldimine making gem–diamine intermediate short lived in solution. Thus, the observed AVA gem–diamine intermediate is likely resulted from a trapping effect during crystal soaking.

It also should be noted that in the soaking structure of AVA, a strong tetrahedral electron density was detected in the proximity of Ser186, Met201, and Phe204 in two of three monomer copies in the asymmetric unit (Fig. S14). Of all chemical species present in crystallization condition, only the phosphate group of PLP possesses the right geometry to fit into the observed electron density. The electron density elongates toward Asp205, but it becomes weaker extending from the tetrahedral center. For this reason, only the phosphoryl group of PLP was built and refined into the observed density. This phosphate-binding site is located at the interface of two monomers that are crystallographically adjacent creating a cavity where PLP could potentially bind. This cavity does not exist in the biological dimer of *h*OAT. In the biological dimer, the Ser186–Met201–Phe204 site is located on the surface of the enzyme, exposed to the bulk solvent. The electron density observed in the Ser186–Met201–Phe204 site likely represents a PLP bound only as a crystallographic artifact. Theoretically, the binding triad of Ser186, Met201, and Phe204 could interact with tetrahedral ligands such as phosphate, in the biological dimer, but it is unclear whether this potential binding site is functional.

## Discussion

Despite that *h*OAT is an important target in cancer therapy, many of its structural and mechanistic properties remain poorly described. In our current work, we address some of the fundamental questions associated with the substrate recognition, catalysis, and pH dependence of *h*OAT. Continued development of rationally designed mechanism-based inactivators of *h*OAT will be facilitated by an expanded understanding of the enzyme's structure and mechanism. Moreover, the discovery of various GABA analogs as potent and specific MBIs of *h*OAT prompted the investigation of the binding of GABA to the *h*OAT active site.

Virtually, the same p*K*a values were determined for the initial and rate-limiting step of both the first and second half-reactions suggesting that these dependencies arise from titration of the same active-site residue. The catalytic residue Lys292 is a candidate residue whose protonation state would critically influence multiple steps of the catalysis. For the first half-reaction, the rate is dependent on the abstraction of the proton from δ-carbon of l-Orn in the external aldimine state by the deprotonated amino group of Lys292. Hence, basic pH facilitates the first half-reaction as Lys292 can more readily act as a general base to form quinonoid (Fig. S1). In the second half-reaction, the rate likely depends on the formation of the PMP–α-KG Schiff base, which requires the amino group on the side chain of Lys292 to be in a protonated state. In the process of the ketimine formation, protonation of Lys292 lowers the p*K*a of the PMP amino group facilitating nucleophilic attack of the α-KG carbonyl. Thus, it is conceivable that in both half-reactions, the protonation state of Lys292 governs the rate-limiting step.

The structure from *h*OAT crystallized at pH 6.0 revealed that the enzyme’s active site has rearranged side-chain conformations that hinder ligand entry to the catalytic pocket. The side chains of Arg180 and Glu235 were found to deviate from their functional positions observed at pH values above neutrality. At low pH, both residues partially occupy the l-Orn binding pocket. Almost no activity was observed with l-Orn at pH 6.6 and lower, but the second half-reaction with α-KG had the maximum reaction rate at pH values below 6.0 and decreased toward higher pH values. Despite the constriction of the active-site pocket at low pH, α-KG is evidently able to react with the PMP form of the enzyme productively. Moreover, this observation may infer a critical role of Arg413 in facilitating the reverse reaction of *h*OAT. It is possible to surmise that once the PMP state is formed and the Arg413–Glu235 salt bridge is broken, the active site has a preference to bind α-KG for the second half-reaction through direct binding interaction with Arg413. Such a conformational constriction would selectively inhibit the first half-reaction and ensures that the enzyme remains in a PLP form at low pH values.

*h*OAT can be found in almost all tissues of the human body, but it predominates in the liver (pH 7.0), kidney (pH 7.4), intestine (pH varies from ∼6.0 to ∼7.4), duodenum (pH 6.0), and retina (pH 7.2) (38, 39, 40, 41). In most cases, *h*OAT is found in tissues where pH is below the determined kinetic p*K*a, in some cases by multiple pH units. In the most acidic conditions, the first half-reaction of *h*OAT with l-Orn would be ostensibly nonfunctioning. It should be noted, however, that in mammals, OAT localizes within the mitochondrial matrix where pH is ∼7.8 (42, 43). Thus, the enzyme is likely less or nonfunctional outside the mitochondria, especially if intracellular pH (pH_i_)/extracellular pH is significantly lower than 7.8. Cancer cells are generally associated with higher pH_i_s of 7.12 to 7.65 compared with pH_i_ 7.0 to 7.2 for the normal cells (44, 45). Such a rise in pH could result in activation of *h*OAT and acceleration of its first half-reaction without significant diminishment of the second half-reaction (Fig. 3). Recently published work suggests that HCC cells are characterized by hydroxyproline accumulation and accelerated consumption of l-proline (19) resulting from an abnormal proline metabolism. At the end of its first half-reaction, *h*OAT produces l-GSA, which cyclizes to form P5C, a precursor to l-proline. Thus, *h*OAT could serve as a regulator of HCC progression *via* the proline metabolic pathway (18). For this reason, the pH dependence of *h*OAT may exacerbate its contribution to HCC progression.

Of the substrates studied, AVA and GABA demonstrated a tighter binding to *h*OAT than l-Orn. The turnover of AVA serves as a proof that the missing α-amino group does not prevent the substrate’s initial binding to the enzyme. In comparison to l-Orn, it appears that the α-amino group plays a crucial role in further catalysis since the observed reaction rate for l-Orn was ∼500 times faster than for AVA. It is also important to note that the first half-reactions with l-Orn and DABA went to completion and did not demonstrate significant reversibility unlike the fractional net conversion to predominant PMP form for the enzyme observed with AVA and GABA. The stopped-flow experiments with AVA showed the dependence of absorbance amplitudes at different ligand concentrations. We propose that the reaction is readily reversible based on the observation that ∼50% of products converted back to reactants when 8 mM of AVA is added (Fig. S15). The reversibility of the reaction could be explained by the missing α-amino group in the structure of AVA. Because of the absence of α-amino group, the reaction product of AVA, 5-oxopentanoic acid, cannot cyclize unlike products from other substrates such as L-GSA and 2-amino-4-oxobutanoic acid. As a result, it can react with PMP to form PLP and AVA. A similar result was observed for GABA; the reversibility of GABA reaction catalyzed by *h*OAT is even more dramatic displaying ∼92% PLP at equilibrium in the presence of 1 mM GABA (based on the observed change in the amplitude of external aldimine at 423 nm).

**Figure 7:**
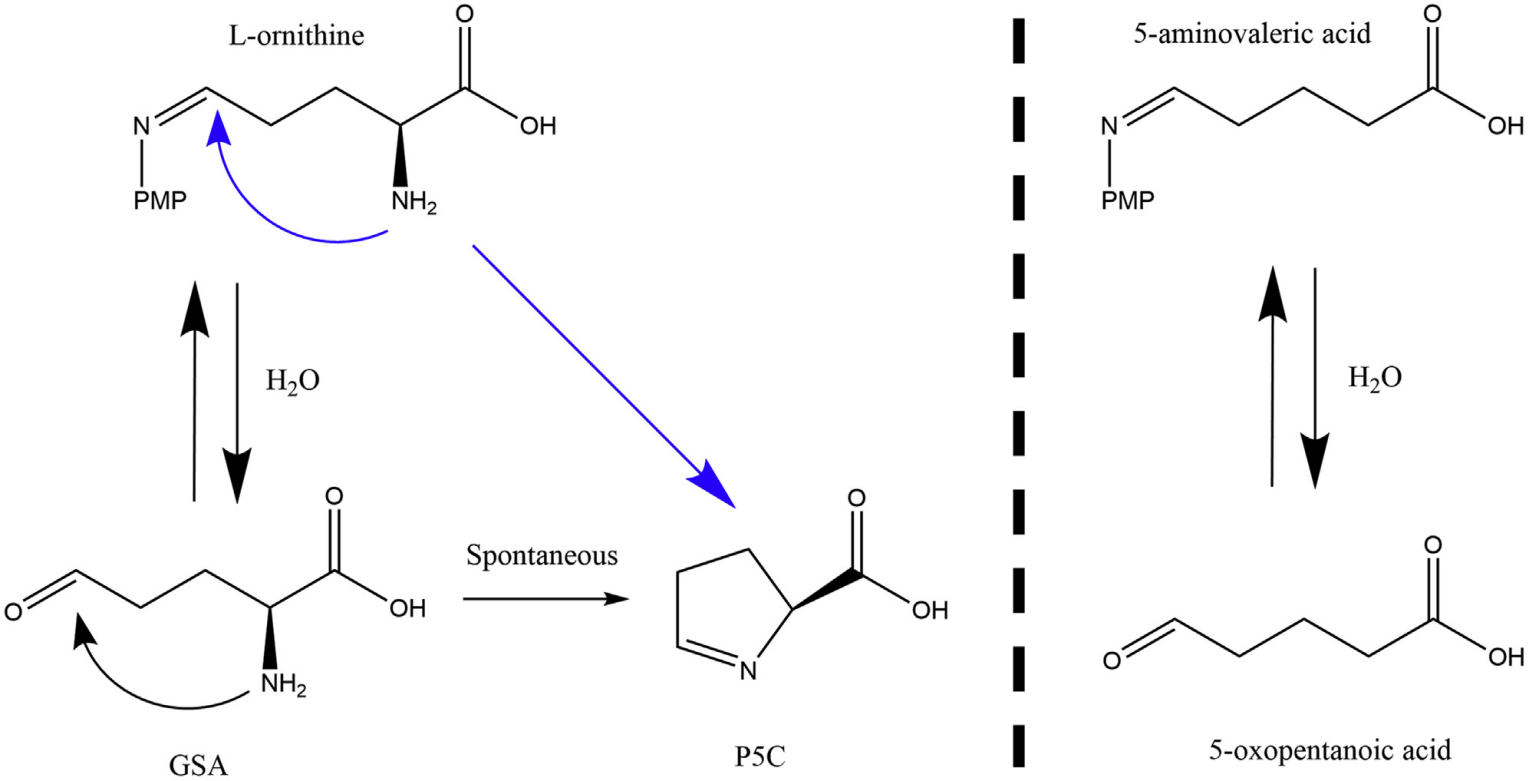
**Potential pathways for cyclization in the last step of the forward half-reaction of *h*OAT (*left*).** AVA, in turn, has only one possible way to dissociate from PMP: through hydrolysis. AVA, 5-aminovaleric acid; *h*OAT, human ornithine aminotransferase; PMP, pyridoxamine phosphate.

The slow reaction rate of AVA could also indirectly support a hypothesis that the last reaction step of *h*OAT may not solely be hydrolysis followed by spontaneous cyclization of the product in solution. Instead, the dissociation of the ligand from PMP could also proceed through 5-exo-trig cyclization initiated by the nucleophilic attack of the α-amino group (Fig. 7) before release. Moreover, we speculate that a faster product release route can be achieved *via* a cyclization step initiated by the α-amino group within the active site by bypassing a hydrolysis step and the formation of GSA. However, our data for *h*OAT with l-Orn indicate that the reaction comes to aldimine–PMP equilibrium at low pH values establishing l-GSA as the product of the reaction and that cyclization to P5C occurs in solution.

For all *h*OAT ligands studied in this work, the transient-state kinetic experiments showed that an early step in all reactions was rate limiting in the first half-reaction. The rate-limiting step corresponding to the decay of a species with an absorbance peak at ∼425 nm likely indicates the conversion of external aldimine. The crystal soaking experiments with GABA and AVA revealed two different stable intermediates. The structure obtained by soaking with GABA could be equally well described by the external aldimine, quinonoid, or ketimine species. However, considering the transient-state data, the external aldimine appears to be the most probable reaction intermediate to be observed in the crystal structure. For AVA, the observed structure correlates with a gem–diamine species. Gem–diamine intermediate found in AVA crystal structure was not detected in the kinetic studies. This could mean that in crystallo the rate-limiting step of *h*OAT reaction with AVA is the decay of the gem–diamine rather than the external aldimine observed in solution. To the best of our knowledge, this is the first experimentally observed gem–diamine structure among all *h*OAT substrates. In the context of *h*OAT, AVA acts as a slow substrate with a binding affinity ∼6.4 times higher than for l-Orn. In fact, its binding affinity is comparable and, in some situations, stronger than those of some known *h*OAT inactivators.

In the current work, structural and kinetic properties of *h*OAT were tested and analyzed. New important information concerning kinetic p*K*a, potential routes for product cyclization, and roles of α-amino and δ-amino groups in substrate recognition and catalysis was obtained. The kinetic and structural results could enhance the foundation for the rational design of a new generation of *h*OAT inactivators. Specifically, the reported results revealed intrinsic information on the active site of nucleophile Lys292, which often forms covalent linkage attacking the electrophilic centers during MBI inactivation (21, 22, 29). In addition, the results also provided a rationale that the potent GABA-mimicking MBIs follow the same binding mode as GABA. This supports the notion that mimicking fragment-sized slow-reacting alternative substrates like GABA could be a more effective approach in structure-based drug design instead of mimicking enzyme’s native substrates (ornithine or glutamate).

As alternative substrates, GABA and AVA displayed potential inhibitory effects against an established drug target *h*OAT because of a combination of stronger binding affinity and slow turnovers. The data for AVA and GABA also lead to a hypothesis of an alternative catalytic mechanism in which a cyclization step bypasses hydrolysis of the ketamine state. Potentially, the “uncyclizable” analogs of GABA could be developed as inhibitory therapeutics against the cancer target *h*OAT if binding selectivity was achieved.

## Experimental procedures

### Expression and purification of *h*OAT

*h*OAT was expressed and purified according to previously published protocols (30). Briefly, *Escherichia coli* BL21(DE3) cells containing the pMAL-t-*h*OAT plasmid were incubated at 37 °C with shaking in lysogeny broth medium supplemented with 100 μg/ml ampicillin. When the culture absorbance value at 600 nm reached a value of 0.7, expression of the MBP−t-*h*OAT fusion protein was induced by the addition of 0.3 mM isopropyl β-d-1-thiogalactopyranoside and incubated for an additional 16 to 18 h at 25 °C. Cells were harvested by centrifugation, washed with buffer A comprised of 20 mM Tris–HCl, 200 mM NaCl, and 100 μM PLP, pH 7.4, frozen in liquid nitrogen, and stored at −80 °C. The frozen cell pellet was then thawed, sonicated in buffer A, and centrifuged at 40,000*g* for 20 min. The resulting supernatant was loaded onto an amylose affinity column pre-equilibrated with buffer A. The column was washed thoroughly, and the MBP−t-*h*OAT fusion protein was eluted from the column with 10 mM maltose. Fractions containing the fusion protein were combined and treated with tobacco etch virus protease to remove the MBP tag. The cleaved *h*OAT protein was collected and concentrated using a centrifugal filter. The protein was then further purified by size-exclusion chromatography using a HiLoad Superdex-200PG column. The column was pre-equilibrated, and the protein was eluted in buffer containing 50 mM Hepes, 100 μM PLP, and 300 mM NaCl, pH 7.5.

### Transient-state experiments for pH studies

To ensure a constant osmotic pressure for experiments that required a range of pH values, a mixture of 50 mM MES, 50 mM acetic acid, 100 mM Tris, and 50 mM NaCl (MAT buffer) was used to buffer for pHs 5.0 to 9.3 (46). For the forward *h*OAT reaction with l-Orn, the enzyme was buffer-exchanged into 1/20 MAT buffer, pH 7.5, and concentrated to 36 μM. A separate 2/1 MAT buffer with 2 mM l-Orn was prepared, and its pH was adjusted to 5.0 using concentrated acetic acid. Solutions with 36 μM *h*OAT and 2 mM l-Orn were mounted into two separate syringes and loaded onto a Hitech Scientific (TgK) stopped-flow spectrophotometer. The absorption changes that occurred when these solutions were combined were observed at all wavelengths from 250 to 800 nm using CCD detection at the temperature of 20 °C. Depending on the pH, the time recorded for each reaction varied in accordance with the observed rate. For pH 5.8, spectra were recorded for 0.001 to 30 and 0.001 to 1600 s; for pH 6.0 to 6.6, spectra were recorded for 0.001 to 30 and 0.001 to 790 s; for pH 6.9 to 7.2, spectra were recorded for 0.001 to 1.5 and 0.001 to 317 s; for pH 7.5 to 7.8, spectra were recorded for 0.001 to 1.5 and 0.001 to 30 s; and for pH 8.1 to 9.0, spectra were collected for 0.001 to 1.5 s. Kinetic data collected on two time frames were spliced together at the limit of the shorter collection period to form composite datasets with time resolution sufficient to describe all events.

For pH studies of the second half-reaction of *h*OAT, the enzyme was buffer-exchanged to 1/20 MAT, pH 7.0 with 2 mM (final) l-Orn, and left to react at 4 °C for 16 h. The PMP form of *h*OAT was then extensively buffer-exchanged to 1/20 MAT, pH 7.0. The protein was further concentrated to 24 μM by centrifugation using 10 kDa molecular weight cutoff filters (Amicon). A separate 2/1 MAT buffer with 2 mM α-KG was prepared, and its pH was adjusted to 5.0 using concentrated acetic acid. Solutions with 24 μM *h*OAT and 2 mM α-KG were placed into two separate syringes and loaded onto the stopped-flow spectrophotometer. The absorption changes were observed at all wavelengths from 250 to 800 nm using a CCD detector at 10 °C. Depending on the pH, the time recorded for each reaction varied in accordance with their observed rates and completeness. For pH 5.0 to 8.7, the spectrum was recorded for 0.001 to 1.5 s; for pH 9.0, 0.001 to 3 s; and for pH 9.3, 0.001 to 8 s. Since reactant ratios establish pseudo–first-order conditions, the obtained data for 420 nm were extracted from the dataset for each pH value and fit to a single exponential to obtain the observed rate constants (Equation 1). In this equation, *k*_obs_ is the observed rate constant, Abs_*t*_ is 420 nm absorbance at time *t*, Abs_end_ is the final absorbance at 420 nm, ΔAbs is the difference between the initial absorbance and the final absorbance (Abs_end_)

(1) $$\mathrm{Ab}s_{t}=\;\Delta \mathrm{Abs}•{\text{e}}^{\left(-k_{\text{obs}}t\right)}+\mathrm{Ab}s_{\text{end}}$$

The pH dependence of *k*_obs_ was fit into Equation 2. *K*_a_ values determined from titratable phenomena X (in this case, *k*_obs_) were determined by plotting the pH against *k*_obs_; where *X*_AH_ and *X*_A_^−^ represent the respective fully protonated and unprotonated arms of the titration.

(2) $$X=\;\frac{\left(X_{\mathrm{AH}}\left[H^{+}\right]+\;K_{\text{a}\;}X_{{\text{A}}^{-}}\right)}{\left[H^{+}\right]+\;K_{\text{a}}}$$

### Transient-state kinetics for *h*OAT substrate analogs

For the stopped-flow experiments, purified *h*OAT was buffer-exchanged into 100 mM Hepes, 50 mM NaCl, pH 7.5 buffer, and concentrated by centrifugation using 10 kDa molecular weight cutoff filters. For the single turnover experiments, three substrate analogs of *h*OAT were chosen: AVA, DABA, and GABA. Equivalent experiments were carried out on l-Orn as a control. For each ligand, a stock solution in 100 mM Hepes, 50 mM NaCl, pH 7.5 was prepared and two-fold serially diluted using the same buffer. Chemical reactions were observed using a stopped-flow spectrophotometer in combination with CCD in a range from 250 to 800 nm. For all reactions, the temperature was held constant at 20 °C. For each set of experiments, *h*OAT and one of the ligands were mounted from two separate syringes onto the stopped-flow instrument where they were rapidly mixed at a 1:1 ratio. For the *h*OAT reaction with l-Orn, the final concentrations after mixing were 9 μM *h*OAT and 16 mM, 8 mM, 4 mM, 2 mM, 1 mM, 500 μM, 250 μM, and 125 μM for l-Orn. For the *h*OAT reaction with AVA, the final concentrations after mixing were 9 μM *h*OAT and 8 mM, 4 mM, 2 mM, 1 mM, 500 μM, 250 μM, and 125 μM AVA. For the *h*OAT reaction with DABA, the final concentrations after mixing were 10.40 μM *h*OAT and 64, 32, 16, 8, and 4 mM DABA. For the *h*OAT reaction with GABA, the final concentrations upon mixing were 9 μM for *h*OAT and 2 mM, 1 mM, 500 μM, 250 μM, and 125 μM GABA. The aforementioned reactant ratios establish pseudo–first-order conditions for all reactions. As such, the data for 335 nm obtained for l-Orn, AVA, and DABA were extracted from the dataset for each and fit to a single exponential to obtain the observed rate constants (Equation 1). In this equation, *k*_obs_ is the observed rate constant, Abs_*t*_ is 335 nm absorbance at time *t*, Abs_end_ is final absorbance at 335 nm, ΔAbs is the difference between the initial absorbance and the final absorbance (Abs_end_).

For each alternative substrate, the rates were plotted against the ligand’s concentration and fit into hyperbolic (l-Orn and AVA; Equation 3) or linear fit (DABA; Equation 4). In Equation 3, *k*_obs_ represents the observed rate constant, *k*_lim_ is the limiting rate constant, [*S*] is the concentration of alternative substrate, and *K*_*d*_ is a dissociation constant. In Equation 4, *k*_obs_ represents observed rate constant, *k*_on_ is association rate constant, [*S*] is the concentration of alternative substrate, and *k*_off_ is the dissociation rate constant.

(3) $$k_{\text{obs}}=\;\frac{\left(k_{\text{lim}}\times \left[S\right]\right)}{\left(K_{d}+\left[S\right]\right)}$$

(4) $$k_{\text{obs}}=\;k_{\text{on}}•\left[S\right]\;+k_{\text{off}}$$

The *K*_*d*_ of GABA was measured in a separate titration experiment. About 14.5 μM (final) *h*OAT in 100 mM Hepes, 50 mM NaCl, pH 7.5 buffer was titrated with 4, 8, 16, 32, 64, 128, 256, 512, and 1024 μM (final) GABA and was allowed to react over 12,000 s. The absorbance spectrum was measured on a UV–Vis spectrophotometer from 250 to 700 nm. From the resulting data, a double-reciprocal plot of 1/[GABA] *versus* 1/ΔAbs at 335 nm was made and fit into Equation 5. ΔAbs_max_ represents the maximum change in absorbance, [GABA] is the concentration of GABA, *K*_*d*_ is the dissociation constant.

(5) $$\frac{1}{\Delta \mathrm{Abs}}=\frac{K_{d}}{\frac{1}{\Delta \mathrm{Ab}s_{\text{max}}}\times \left[\text{GABA}\right]}+\;\frac{1}{\Delta \mathrm{Ab}s_{\text{max}}}$$

The GABA concentration was plotted against the saturated fraction of *h*OAT and fitted into a hyperbolic curve (Equation 6). In Equation 6, *f*_max_ represents a fraction of fully saturated enzyme, [GABA] is the concentration of GABA, and *K*_*d*_ is the dissociation constant.

(6) $$f=\;\frac{\left(f_{\text{max}}\times \left[\text{GABA}\right]\right)}{\left(K_{d}+\left[\text{GABA}\right]\right)}$$

### *h*OAT holoenzyme pH 6.0 crystallization

Purified holo-*h*OAT was buffer-exchanged into 100 mM MES, 200 mM NaCl, 100 μM PLP, pH 6.0 buffer, and then concentrated to ∼6 mg/ml. The crystallization was performed *via* the hanging-drop vapor diffusion method according to previously published conditions with 50 mM tricine (pH 7.8) substituted to 50 mM MES (pH 6.0) buffer. The crystals grew at room temperature within 3 days and reached their maximum size in a week. The crystals had cubic morphology with the largest dimension of ∼0.3 mm. Once no further growth of crystals was observed, they were transferred into cryoprotectant solution (well solution + 30% glycerol) and flash-frozen in liquid nitrogen.

### *h*OAT crystal soaking with GABA and AVA

Once *h*OAT was purified, it was transferred to a 10 kDa centrifugal filter tube and concentrated to ∼6 mg/ml. The holoenzyme crystals were first grown *via* a hanging-drop vapor diffusion method. Each drop contained 2 μl of protein and 2 μl of well solution. The best crystallization condition contained 8% PEG 6000, 100 mM NaCl, 5% glycerol, and 50 mM tricine (pH 7.8). Once holoenzyme crystals reached their maximum size within 7 days, 1 μl of 10 mM GABA or AVA was added to the drop with crystals. The crystals were soaked for different periods from 3 to 59 min for AVA and from 50 min to 2.5 h for GABA. After soaking, crystals were transferred into a cryoprotective solution (well solution supplemented with 30% glycerol) and then flash-frozen in liquid nitrogen.

### X-ray diffraction and data processing

Monochromatic X-ray diffraction data were collected at the LS-CAT beamline 21-ID-D at the Advanced Photon Source at Argonne National Laboratory. Data were collected at a wavelength of 1.127 Å and a temperature of 100 K using a Dectris Eiger 9M detector. Datasets were processed and analyzed with autoPROC (47) or iMosflm (48) software.

### Model building and refinement

The *h*OAT structure was solved by molecular replacement using PHASER (49) in Phenix (50). The starting search model was the previously published structure of *h*OAT (PDB code: 1OAT). The model building and refinement were accomplished in Coot (51) and Phenix, respectively, as an iterative process until the lowest possible *R*_free_*/R*_work_ factor values were attained. Structural depiction figures were prepared using UCSF Chimera (52).

## Data availability

Atomic coordinates and corresponding structure factors have been deposited at the PDB as 7T9Z for *h*OAT crystallized at pH 6.0, 7TA0 for *h*OAT soaked with AVA, and 7TA1 for *h*OAT soaked with GABA. Authors will release the atomic coordinates upon article publication.

## Supporting information

This article contains supporting information.

## Conflict of interest

The authors declare that they have no conflicts of interest with the contents of this article.

## References

[1] Herzfeld A., Knox W.E. (1968). The properties, developmental formation, and estrogen induction of ornithine aminotransferase in rat tissues. J. Biol. Chem..

[2] Wang T., Lawler A.M., Steel G., Sipila I., Milam A.H., Valle D. (1995). Mice lacking ornithine–δ–amino–transferase have paradoxical neonatal hypoornithinaemia and retinal degeneration. Nat. Genet..

[3] Wu G., Knabe D.A., Kim S.W. (2004). Arginine nutrition in neonatal pigs. J. Nutr..

[4] Tomlinson C., Rafii M., Sgro M., Ball R.O., Pencharz P. (2011). Arginine is synthesized from proline, not glutamate, in enterally fed human preterm neonates. Pediatr. Res..

[5] Kohler E.S., Sankaranarayanan S., van Ginneken C.J., van Dijk P., Vermeulen J.L., Ruijter J.M., Lamers W.H., Bruder E. (2008). The human neonatal small intestine has the potential for arginine synthesis; developmental changes in the expression of arginine-synthesizing and -catabolizing enzymes. BMC Dev. Biol..

[6] Montioli R., Bellezza I., Desbats M.A., Borri Voltattorni C., Salviati L., Cellini B. (2021). Deficit of human ornithine aminotransferase in gyrate atrophy: Molecular, cellular, and clinical aspects. Biochim. Biophys. Acta (Bba) - Proteins Proteomics.

[7] Ramesh V., Gusella J.F., Shih V.E. (1991). Molecular pathology of gyrate atrophy of the choroid and retina due to ornithine aminotransferase deficiency. Mol. Biol. Med..

[8] Colnot S., Decaens T., Niwa-Kawakita M., Godard C., Hamard G., Kahn A., Giovannini M., Perret C. (2004). Liver-targeted disruption of <em>Apc</em> in mice activates β-catenin signaling and leads to hepatocellular carcinomas. Proc. Natl. Acad. Sci. U. S. A..

[9] Cadoret A., Ovejero C., Terris B., Souil E., Levy L., Lamers W.H., Kitajewski J., Kahn A., Perret C. (2002). New targets of β-catenin signaling in the liver are involved in the glutamine metabolism. Oncogene.

[10] Yang J.D., Roberts L.R. (2010). Hepatocellular carcinoma: A global view. Nat. Rev. Gastroenterol. Hepatol..

[11] Sherman M., Bruix J., Porayko M., Tran T., for the A.P.G.C. (2012). Screening for hepatocellular carcinoma: The rationale for the American association for the study of liver diseases recommendations. Hepatology.

[12] Personeni N., Rimassa L. (2017). Hepatocellular carcinoma: A global disease in need of individualized treatment strategies. J. Oncol. Pract..

[13] Sayiner M., Golabi P., Younossi Z.M. (2019). Disease burden of hepatocellular carcinoma: A global perspective. Dig. Dis. Sci..

[14] de Lope C.R., Tremosini S., Forner A., Reig M., Bruix J. (2012). Management of HCC. J. Hepatol..

[15] Milgrom D.P., Maluccio M.A., Koniaris L.G. (2016). Management of hepatocellular carcinoma (HCC). Curr. Surg. Rep..

[16] de Rosamel L., Blanc J.-F. (2017). Emerging tyrosine kinase inhibitors for the treatment of hepatocellular carcinoma. Expert Opin. Emerg. Drugs.

[17] Leathers J.S., Balderramo D., Prieto J., Diehl F., Gonzalez-Ballerga E., Ferreiro M.R., Carrera E., Barreyro F., Diaz-Ferrer J., Singh D., Mattos A.Z., Carrilho F., Debes J.D. (2019). Sorafenib for treatment of hepatocellular carcinoma: A survival analysis from the south American liver research network. J. Clin. Gastroenterol..

[18] Zigmond E., Ben Ya'acov A., Lee H., Lichtenstein Y., Shalev Z., Smith Y., Zolotarov L., Ziv E., Kalman R., Le H.V., Lu H., Silverman R.B., Ilan Y. (2015). Suppression of hepatocellular carcinoma by inhibition of overexpressed ornithine aminotransferase. ACS Med. Chem. Lett..

[19] Ginguay A., Cynober L., Curis E., Nicolis I. (2017). Ornithine aminotransferase, an important glutamate-metabolizing enzyme at the crossroads of multiple metabolic pathways. Biology.

[20] Peraino C. (1972). Functional properties of ornithine-ketoacid aminotransferase from rat liver. Biochim. Biophys. Acta (Bba) - Enzymol..

[21] Butrin A., Beaupre B.A., Kadamandla N., Zhao P., Shen S., Silverman R.B., Moran G.R., Liu D. (2021). Structural and kinetic analyses reveal the dual inhibition modes of ornithine aminotransferase by (1S,3S)-3-Amino-4-(hexafluoropropan-2-ylidenyl)-cyclopentane-1-carboxylic acid (BCF3). ACS Chem. Biol..

[22] Shen S., Butrin A., Doubleday P.F., Melani R.D., Beaupre B.A., Tavares M.T., Ferreira G.M., Kelleher N.L., Moran G.R., Liu D., Silverman R.B. (2021). Turnover and inactivation mechanisms for (S)-3-Amino-4,4-difluorocyclopent-1-enecarboxylic acid, a selective mechanism-based inactivator of human ornithine aminotransferase. J. Am. Chem. Soc..

[23] Lide D.R. (1999).

[24] Hayashi H., Kagamiyama H. (1997). Transient-state kinetics of the reaction of aspartate aminotransferase with aspartate at low pH reveals dual routes in the Enzyme−Substrate association process. Biochemistry.

[25] Shen B.W., Hennig M., Hohenester E., Jansonius J.N., Schirmer T. (1998). Crystal structure of human recombinant ornithine aminotransferase. J. Mol. Biol..

[26] Jansonius J.N., Genovesio-Taverne J.-C., Hennig M., Hohenester E., Jenny M., Malashkevich V., Moser M., Muller R., Shen B.W., Stark W., Stosch V.A., Toney M.D., Marino G., Sannia G., Bossa F. (1994). Biochemistry of Vitamin B6 and PQQ.

[27] Schiroli, D., and Peracchi, A. A Subfamily of PLP-dependent Enzymes Specialized in Handling Terminal Amines. 10.1016/j.bbapap.2015.02.023 25770684

[28] Zhu W., Doubleday P.F., Butrin A., Weerawarna P.M., Melani R.D., Catlin D.S., Dwight T.A., Liu D., Kelleher N.L., Silverman R.B. (2021). Remarkable and unexpected mechanism for (S)-3-Amino-4-(difluoromethylenyl)cyclohex-1-ene-1-carboxylic acid as a selective inactivator of human ornithine aminotransferase. J. Am. Chem. Soc..

[29] Zhu W., Doubleday P.F., Catlin D.S., Weerawarna P.M., Butrin A., Shen S., Wawrzak Z., Kelleher N.L., Liu D., Silverman R.B. (2020). A remarkable difference that one fluorine atom confers on the mechanisms of inactivation of human ornithine aminotransferase by two cyclohexene analogues of γ-aminobutyric acid. J. Am. Chem. Soc..

[30] Mascarenhas R., Le H.V., Clevenger K.D., Lehrer H.J., Ringe D., Kelleher N.L., Silverman R.B., Liu D. (2017). Selective targeting by a mechanism-based inactivator against pyridoxal 5′-phosphate-dependent enzymes: Mechanisms of inactivation and alternative turnover. Biochemistry.

[31] Moschitto M.J., Doubleday P.F., Catlin D.S., Kelleher N.L., Liu D., Silverman R.B. (2019). Mechanism of inactivation of ornithine aminotransferase by (1S,3S)-3-Amino-4-(hexafluoropropan-2-ylidenyl)cyclopentane-1-carboxylic acid. J. Am. Chem. Soc..

[32] Williams J.A., Bridge G., Fowler L.J., John R.A. (1982). The reaction of ornithine aminotransferase with ornithine. Biochem. J..

[33] Markova M., Peneff C., Hewlins M.J., Schirmer T., John R.A. (2005). Determinants of substrate specificity in omega-aminotransferases. J. Biol. Chem..

[34] Mitchell G.A., Brody L.C., Sipila I., Looney J.E., Wong C., Engelhardt J.F., Patel A.S., Steel G., Obie C., Kaiser-Kupfer M. (1989). At least two mutant alleles of ornithine delta-aminotransferase cause gyrate atrophy of the choroid and retina in Finns. Proc. Natl. Acad. Sci. U. S. A..

[35] Khersonsky O., Roodveldt C., Tawfik D.S. (2006). Enzyme promiscuity: Evolutionary and mechanistic aspects. Curr. Opin. Chem. Biol..

[36] Rothman S.C., Kirsch J.F. (2003). How does an enzyme evolved *in vitro* compare to naturally occurring homologs possessing the targeted function? Tyrosine aminotransferase from aspartate aminotransferase. J. Mol. Biol..

[37] Storici P., Capitani G., Müller R., Schirmer T., Jansonius J.N. (1999). Crystal structure of human ornithine aminotransferase complexed with the highly specific and potent inhibitor 5-fluoromethylornithine111Edited by R. Huber. J. Mol. Biol..

[38] Dreffs A., Henderson D., Dmitriev A.V., Antonetti D.A., Linsenmeier R.A. (2018). Retinal pH and acid regulation during metabolic acidosis. Curr. Eye Res..

[39] Fallingborg J. (1999). Intraluminal pH of the human gastrointestinal tract. Danish Med. Bull..

[40] Hamm L.L., Nakhoul N., Hering-Smith K.S. (2015). Acid-base homeostasis. Clin. J. Am. Soc. Nephrol..

[41] Park R., Leach W.J., Arieff A.I. (1979). Determination of liver intracellular pH *in vivo* and its homeostasis in acute acidosis and alkalosis. Am. J. Physiol.-Renal Physiol..

[42] Peraino C., Pitot H.C. (1963). Ornithine-δ-transaminase in the rat I. Assay and some general properties. Biochim. Biophys. Acta (Bba) - Specialized Sect. Enzymol. Subj..

[43] Mueckler M.M., Himeno M., Pitot H.C. (1982). *In vitro* synthesis and processing of a precursor to ornithine aminotransferase. J. Biol. Chem..

[44] Hao G., Xu Z.P., Li L. (2018). Manipulating extracellular tumour pH: An effective target for cancer therapy. RSC Adv..

[45] Shirmanova M.V., Druzhkova I.N., Lukina M.M., Matlashov M.E., Belousov V.V., Snopova L.B., Prodanetz N.N., Dudenkova V.V., Lukyanov S.A., Zagaynova E.V. (2015). Intracellular pH imaging in cancer cells *in vitro* and tumors *in vivo* using the new genetically encoded sensor SypHer2. Biochim. Biophys. Acta (BBA)-General Subj..

[46] Ellis K.J., Morrison J.F. (1982). Buffers of constant ionic strength for studying pH-dependent processes. Methods Enzymol.

[47] Vonrhein C., Flensburg C., Keller P., Sharff A., Smart O., Paciorek W., Womack T., Bricogne G. (2011). Data processing and analysis with the autoPROC toolbox. Acta Crystallogr. Sect. D.

[48] Battye T.G.G., Kontogiannis L., Johnson O., Powell H.R., Leslie A.G.W. (2011). iMOSFLM: A new graphical interface for diffraction-image processing with MOSFLM. Acta Crystallogr. D Biol. Crystallogr..

[49] McCoy A.J., Grosse-Kunstleve R.W., Adams P.D., Winn M.D., Storoni L.C., Read R.J. (2007). Phaser crystallographic software. J. Appl. Crystallogr..

[50] Liebschner D., Afonine P.V., Baker M.L., Bunkoczi G., Chen V.B., Croll T.I., Hintze B., Hung L.-W., Jain S., McCoy A.J., Moriarty N.W., Oeffner R.D., Poon B.K., Prisant M.G., Read R.J. (2019). Macromolecular structure determination using X-rays, neutrons and electrons: Recent developments in Phenix. Acta Crystallogr. Sect. D.

[51] Emsley, P., Lohkamp B Fau - Scott, W. G., Scott Wg Fau - Cowtan, K., and Cowtan, K. Features and Development of Coot. 10.1107/S0907444910007493 PMC2852313 20383002

[52] Pettersen, E. F., Goddard Td Fau - Huang, C. C., Huang Cc Fau - Couch, G. S., Couch Gs Fau - Greenblatt, D. M., Greenblatt Dm Fau - Meng, E. C., Meng Ec Fau - Ferrin, T. E., and Ferrin, T. E. UCSF Chimera-Aa Visualization System for Exploratory Research and Analysis. 10.1002/jcc.20084 15264254

