# Supplementary material for: Determination of the pH dependence, substrate specificity, and turnovers of alternative substrates for human ornithine aminotransferase
Source: J Biol Chem. 2022 Apr 20;298(6):101969. doi: 10.1016/j.jbc.2022.101969 (PMC9136103; doi:10.1016/j.jbc.2022.101969)
Supplement: Supplemental Figures S1–S15 and Table S1 [file mmc1.docx]

**Supporting Information**

**Determination of the pH-Dependence, Substrate Specificity and Turnovers of Alternative Substrates for Human Ornithine Aminotransferase**

Arseniy Butrin^1^, Anastassiya Butrin^1^, Zdzislaw Wawrzak^2^, Graham Moran^1^*, and Dali Liu^1^*

^1^ Department of Chemistry and Biochemistry, 1068 W Sheridan Rd, Loyola University Chicago, Chicago, IL 60660

^2^ Synchrotron Research Center, Life Sciences Collaborative Access Team, Northwestern University, Argonne, Illinois 60439, United States

*Corresponding authors; phone: (773)508-3093; email: [dliu@luc.edu](mailto:dliu@luc.edu)

phone: (773)508-3756; email: [gmoran3@luc.edu](mailto:gmoran3@luc.edu)

_
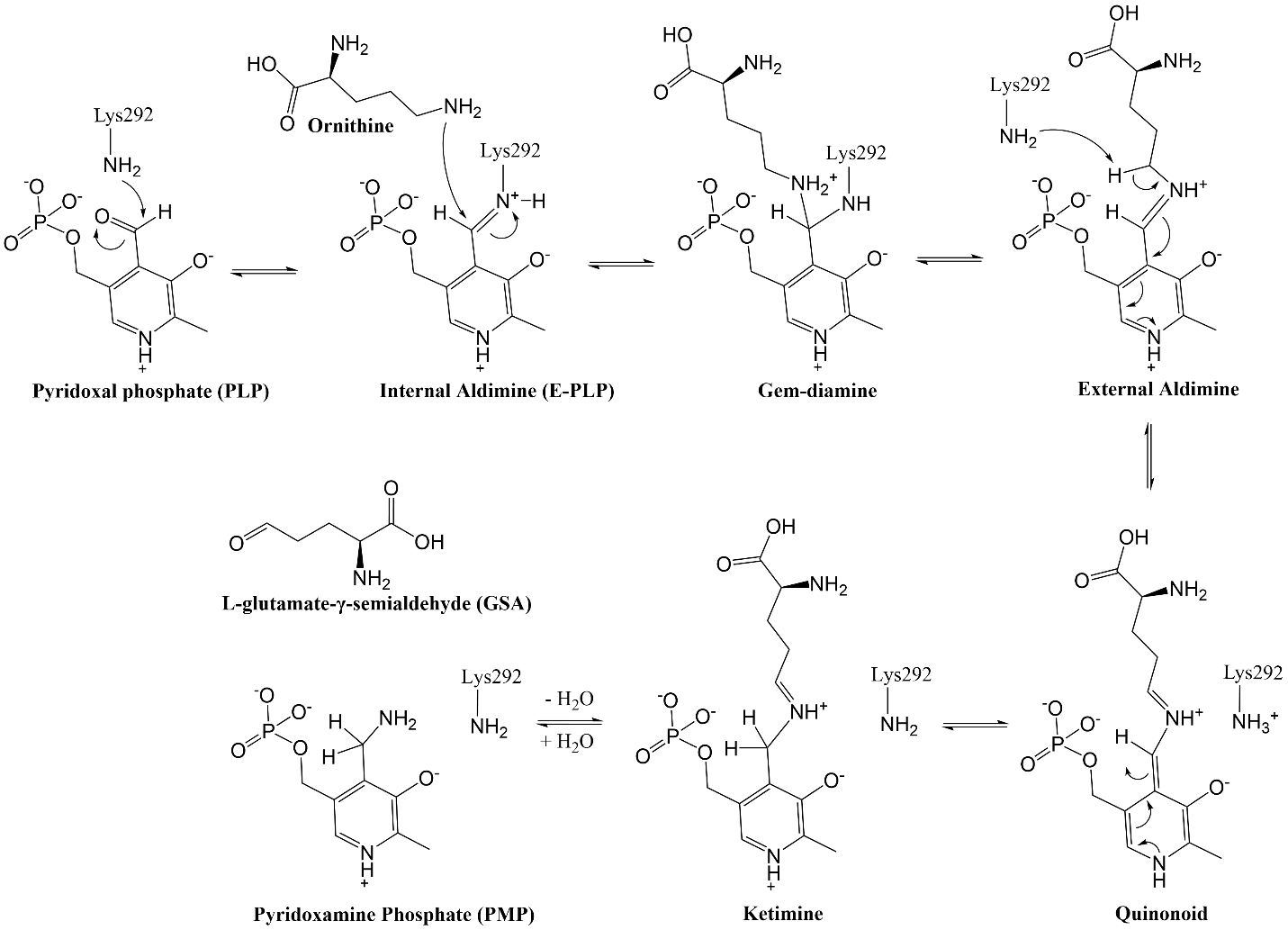
_

**Figure S1. Simplified mechanism of reaction of *h*OAT with its native substrate L-ornithine.**


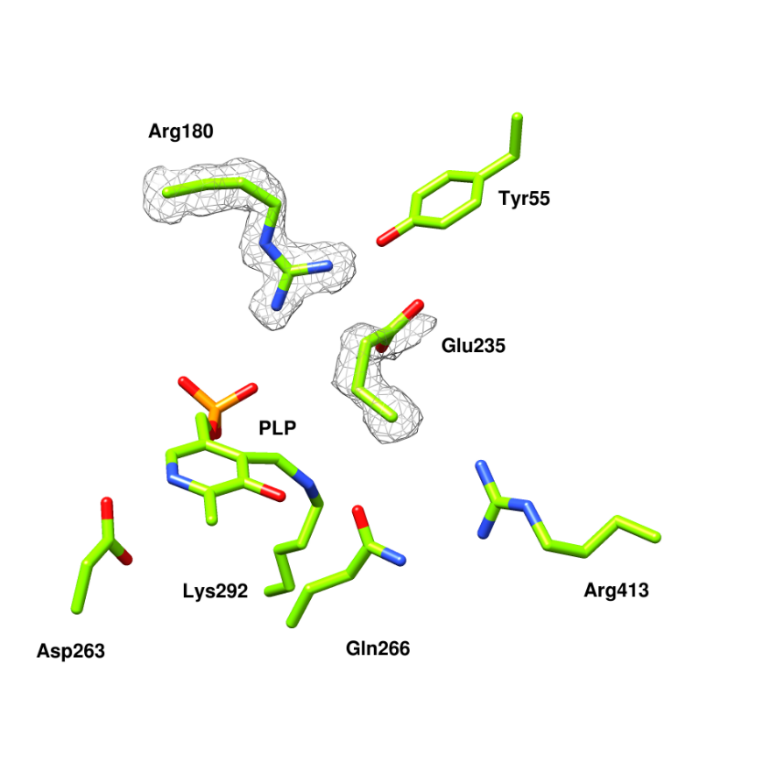


**Figure S2. Polder map (at 2.0 σ level) of *h*OAT crystallized at pH 6.0.** Electron density is shown as gray mesh around Arg180 and Glu235.

| Complex | *h*OAT: holoenzyme, pH 6.0 | *h*OAT:  AVA soaking | *h*OAT:  GABA soaking |
| --- | --- | --- | --- |
| PDB code | 7T9Z | 7TA0 | 7TA1 |
| Space group | C 1 2 1 | P 3_2_ 2 1 | P 3_1_ 2 1 |
| **Cell dimension** | | | |
| α, β, γ (deg) | 90.0, 106.4, 90.0 | 90.0, 90.0, 120.0 | 90.0, 90.0, 120.0 |
| a, b, c (Å) | 200.9, 110.8, 56.9 | 114.9, 114.9, 185.1 | 114.6, 114.6, 349.3 |
| Processed Resolution (Å) | 2.15 | 2.33 | 2.20 |
| Rmerge ^a^ (%) | 16.9 (121.4) | 48.7 (524.4) | 18.7 (182.4) |
| Rpim ^c^ (%) | 10.3 (73.8) | 12.8 (135.3) | 8 (84.1) |
| I/σ (I) | 4.9 (1.2) | 7.0 (0.8) | 6.7 (1.0) |
| CC ½ ^d^ (%) | 98.6 (37.1) | 99.4 (33.0) | 99.3 (41.7) |
| Completeness (%) | 92.5 (90.2) | 99.9 (100.0) | 99.8 (99.9) |
| Multiplicity | 3.4 (3.4) | 15.3 (16.0) | 6.1 (5.4) |
| No. Reflections | 258822 (14325) | 939082 (47999) | 1075016 (137321) |
| No. Unique Reflections | 75968 (4201) | 61264 (2995) | 177531 (25577) |
| **Refinement** | | | |
| Rwork^e^/Rfree^f^ (%) | 27.6/28.0 | 21.3/23.5 | 25.3/27.1 |
| No. of Atoms | | | |
| protein | 9458 | 9440 | 18951 |
| ligand | 45 | 106 | 118 |
| water | 576 | 325 | 896 |
| B factors (Å^2^) | | | |
| protein | 23.0 | 45.0 | 39.0 |
| ligand | N/A^h^ | 44.9 | 25.6 |
| RMSD ^g^ | | | |
| bond lengths (Å) | 0.66 | 0.55 | 0.82 |
| bond angles (deg) | 0.006 | 0.002 | 0.005 |
| **Ramachandran plot (%)** | | | |
| favored | 97.0 | 95.0 | 95.0 |
| allowed | 3.0 | 4.9 | 4.8 |
| outliers | 0.0 | 0.1 | 0.2 |
| ^a^R_merge_ = Σ\|I_obs_ − I_avg_\|/ΣI_avg_  ^b^The values for the highest-resolution bin are in parentheses  ^c^Precision-indicating merging R  ^d^Pearson correlation coefficient of two “half” data sets  ^e^R_work_ = Σ\|F_obs_ − F_calc_\|/ΣF_obs_  ^f^Five percent of the reflection data were selected at random as a test set, and only these data were used to calculate R_free_  ^g^Root-mean square deviation  ^h^Not applicable | | | |

**Table S1. Statistics of the crystal structures of holoenzyme *h*OAT crystallized at pH 6.0, soaked with AVA, and soaked with GABA.**


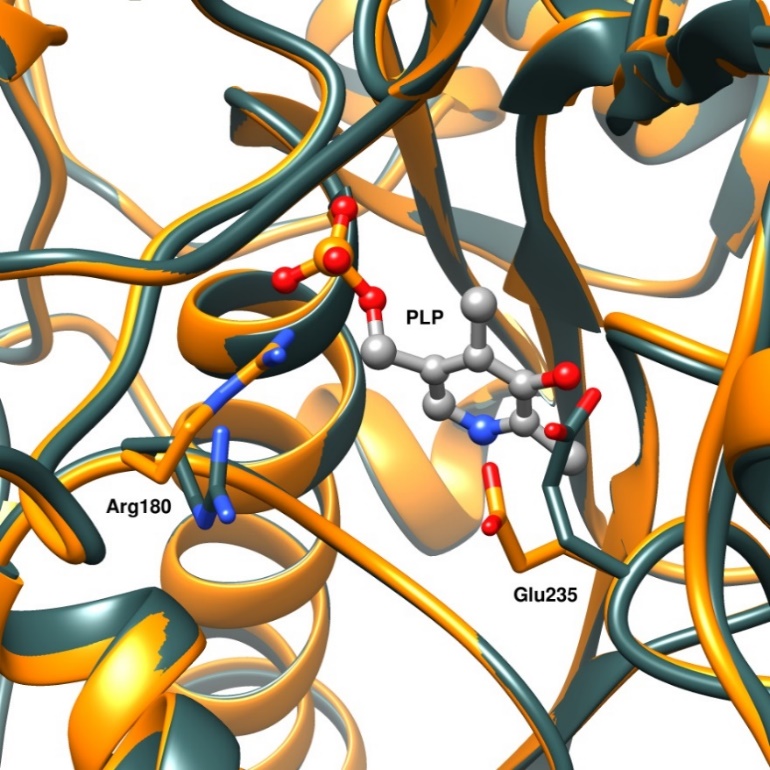


**Figure S3. Overlay of crystal structures of *h*OAT at pH 6.0 (orange) and pH 7.8 (dark blue).** Arg180 and Glu235 side chains are shown as sticks for both structures. The positions of these two residues control the width of the active site pocket.


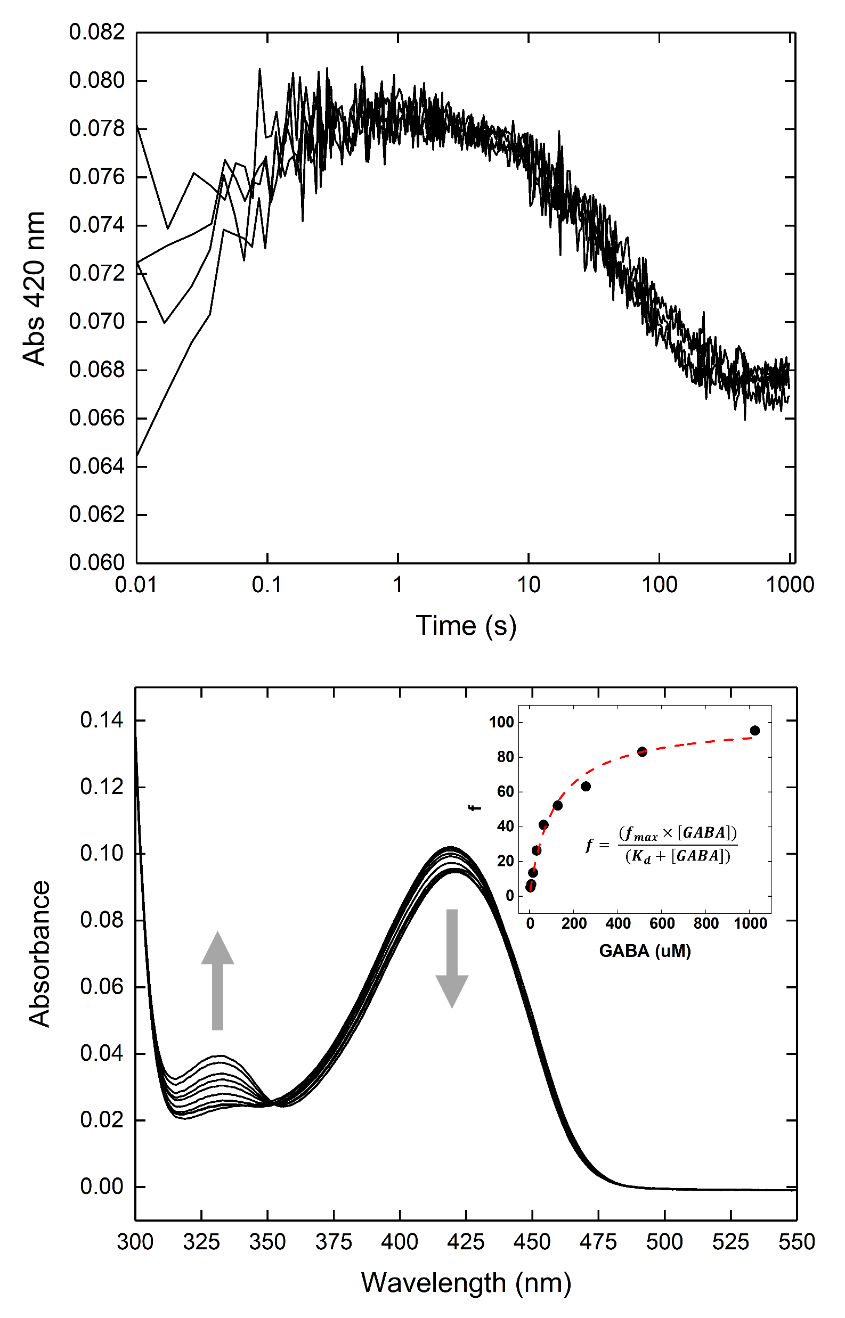


**Figure S4. Top: Transient-state absorption changes observed at 420 nm for 9 µM (final) *h*OAT reacting with 250, 500, 1000, and 2000 µM (final) GABA. Bottom: The binding assay of 14.5 µM (final) *h*OAT with 4, 8, 16, 32, 64, 128, 256, 512, 1024 µM (final) GABA.** In the top right corner is shown dependence of enzyme’s fractional saturation from GABA concentration, the data were fit into hyperbolic curve. The direction of the gray arrow indicates the trend of GABA concentration change towards higher values.





**Figure S5. CCD spectra of *h*OAT (9 µM final) and L-orn (8 mM final) reaction measured within 250-800 nm range.** The spectra were collected at a 0.002-1.97 s timeframe.





**Figure S6. CCD spectra of *h*OAT (9 µM final) and AVA (8 mM final) reaction measured within 250-800 nm range.** The spectra were collected at two timeframes (0.002-1.97, 2.4-394 s) and spliced together at 2.4 s to form a single data set for analysis.





**Figure S7. CCD spectra of *h*OAT (10.4 µM final) and DABA (64 mM final) reaction measured within 250-800 nm range.** The spectra were collected at two timeframes (0.006-9.841, 9.896-394 s) and spliced together at 9.896 s to form a single data set for analysis.





**Figure S8. CCD spectra of *h*OAT (9 µM final) and GABA (16 mM final) reaction measured within 250-800 nm range.** The spectra were collected at two timeframes (0.007-9.842, 10.296-197 s) and spliced together at 10.296 s to form a single data set for analysis.





**Figure S

9. Comparison of the *h*OAT holoenzyme spectrum with the initial spectrum after addition of the 2 mM GABA (top), 1 mM AVA (middle), or 8 mM DABA (bottom).**


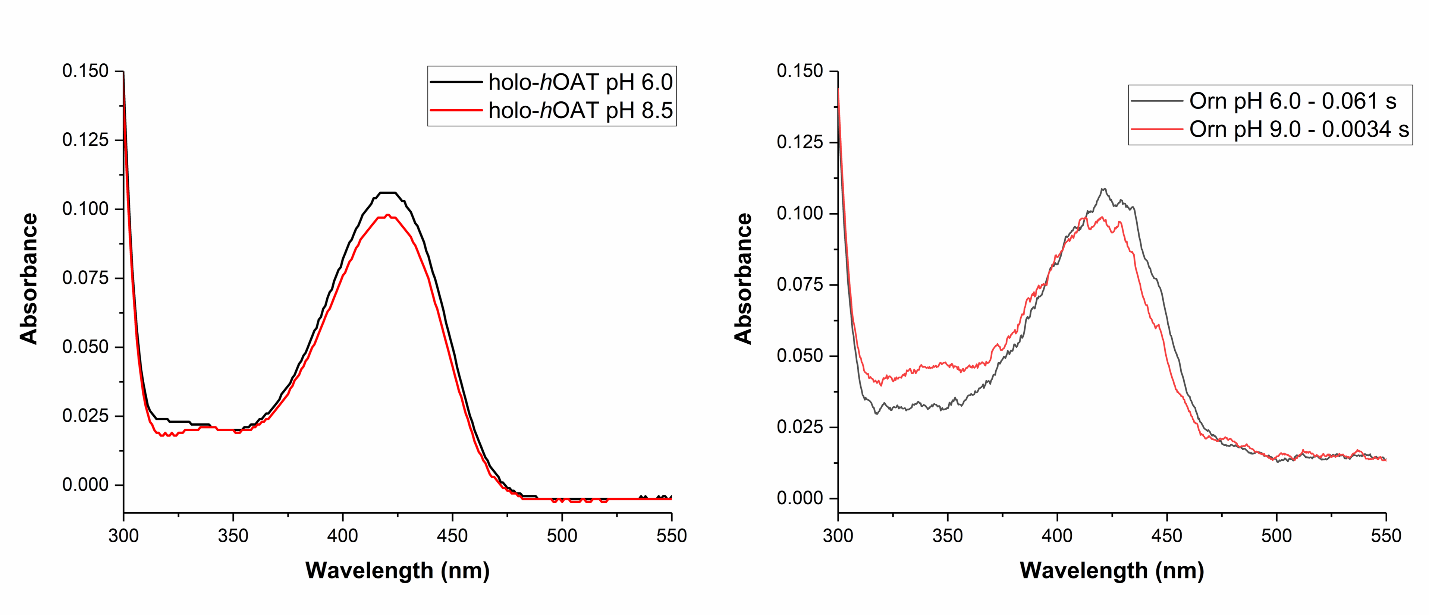


**Figure S10. The absorption spectra of 9 µM holoenzyme *h*OAT at pH 6.0 and pH 8.5 (left).** Initial absorption spectra of the reaction between 36 µM *h*OAT and 2 mM L-ornithine at pH 6.0 and pH 9.0.


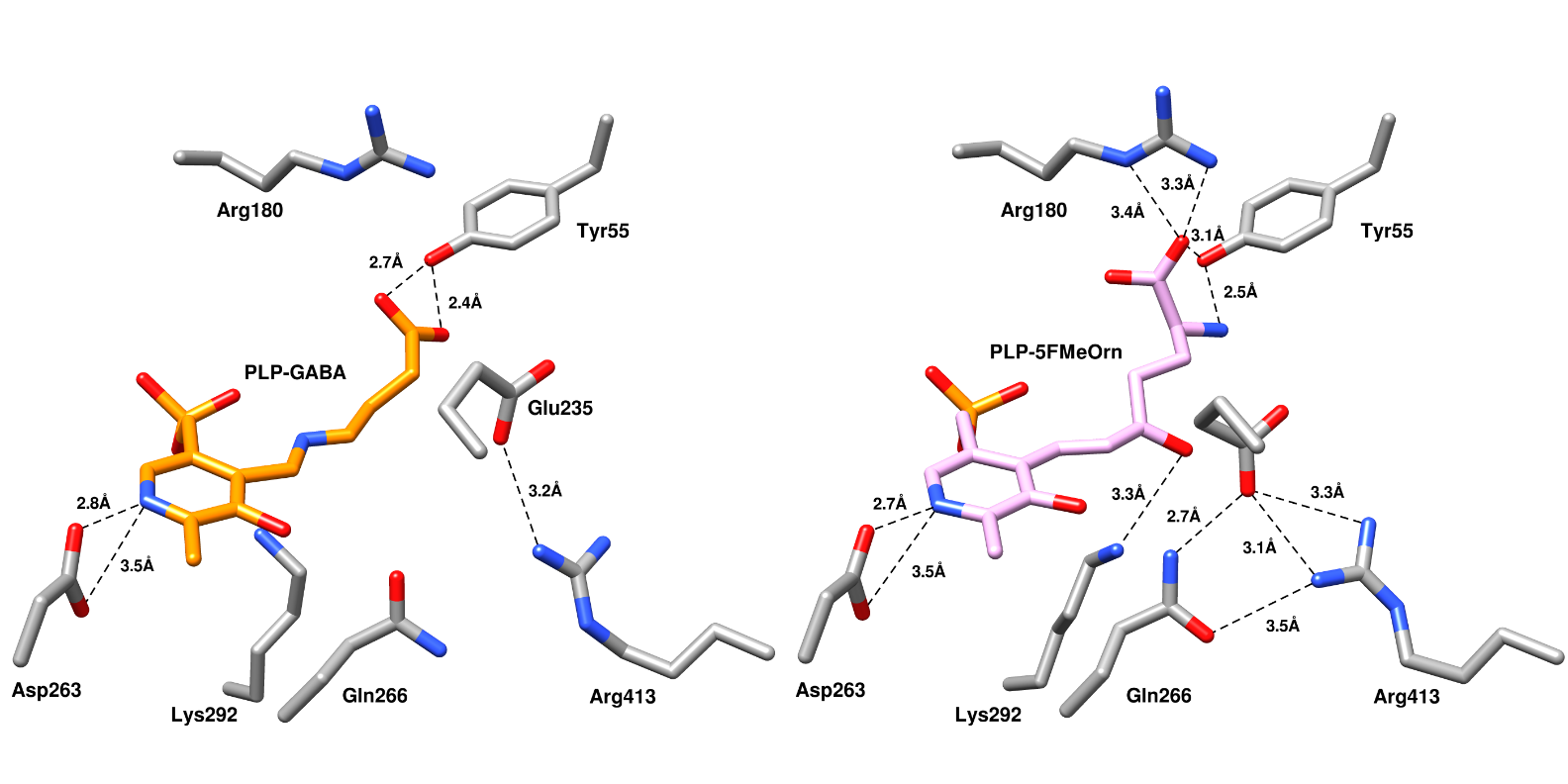
**Figure S11.** Comparison of the *h*OAT GABA derived external aldimine to the final adduct formed in reaction between *h*OAT and its inhibitor 5-fluoromethylornithine (5FMeOrn). Left: active site of *h*OAT bound with external aldimine intermediate trapped during reaction with GABA. Right: Active site of *h*OAT with 5FMeOrn inactivation product. Interactions are shown as black dashed lines.


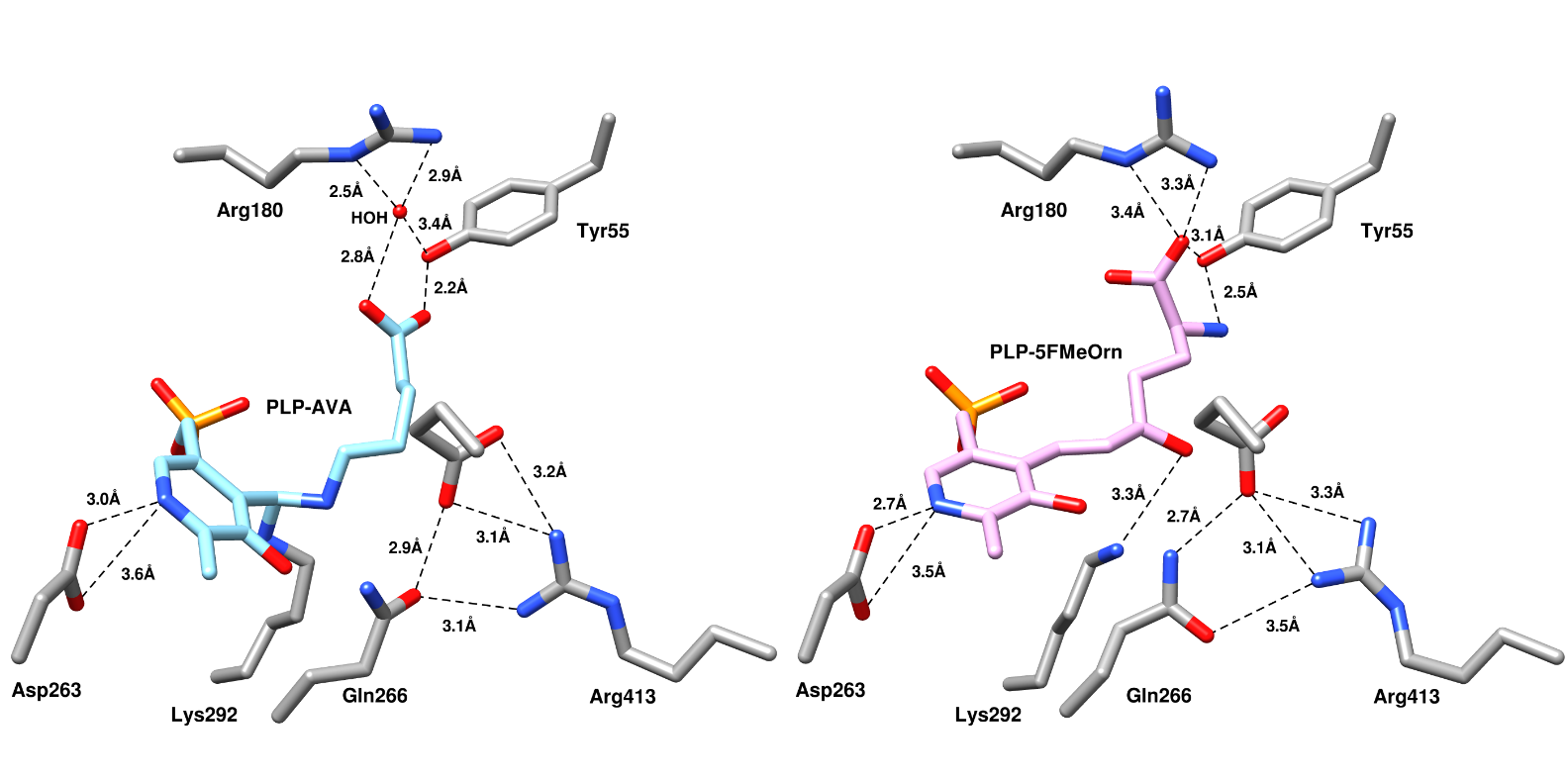
 **Figure S12.** Comparison of the *h*OAT AVA derived gem-diamine to the final adduct formed in reaction between *h*OAT and its inhibitor 5-fluoromethylornithine (5FMeOrn). Left: active site of *h*OAT bound with gem-diamine intermediate trapped during reaction with AVA. Right: Active site of *h*OAT with 5FMeOrn inactivation product. Interactions are shown as black dashed lines.


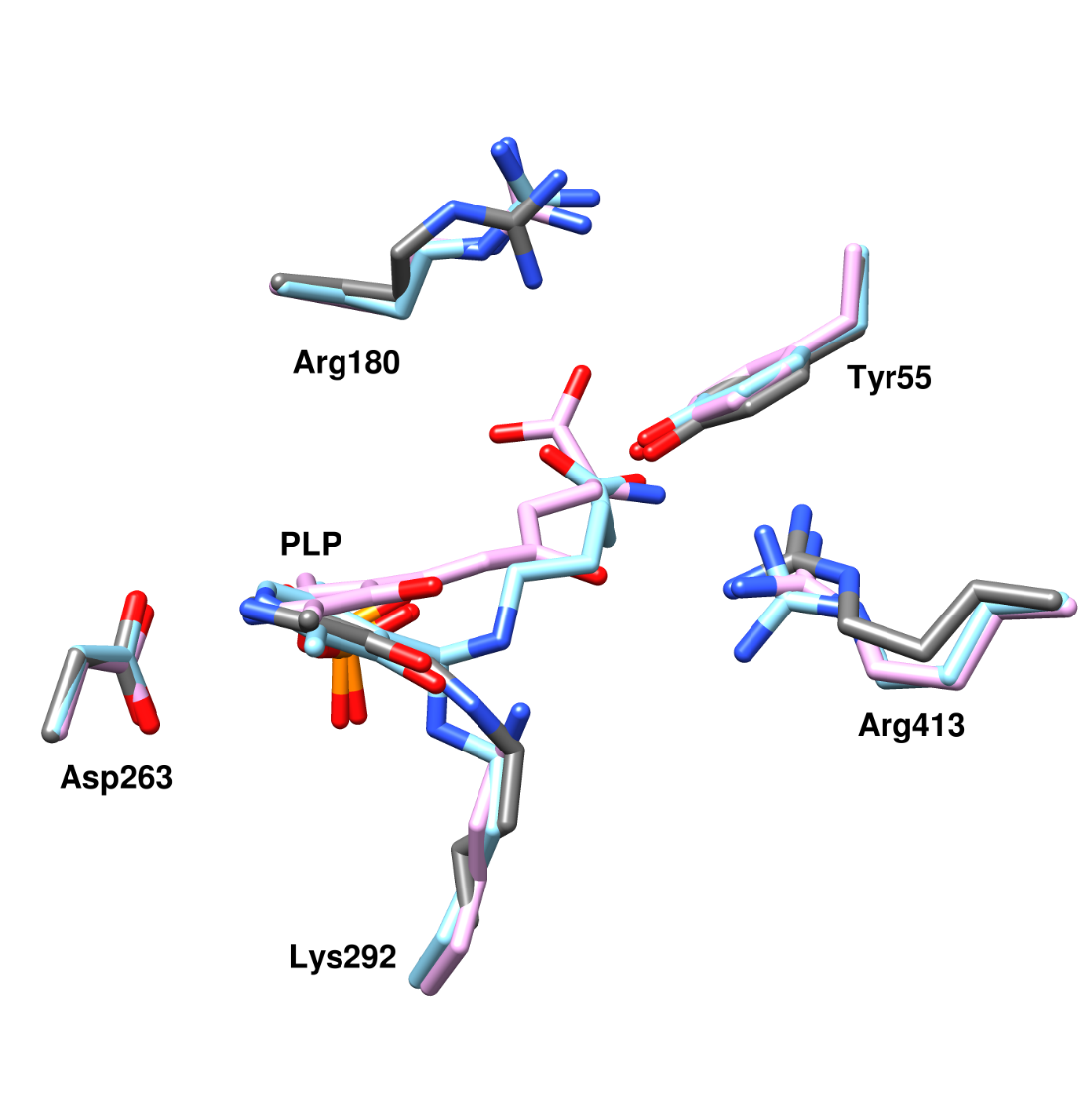


**Figure S13. Comparison of the internal aldimine (gray; PDB ID: 1OAT), gem-diamine of AVA (blue; PDB ID: 7TA0), and external aldimine formed from 5-FMeOrn (pink; PDB ID: 2OAT).** The overlap of these structures shows how orientation of the PLP in internal aldimine and gem-diamine is significantly different from the external aldimine.


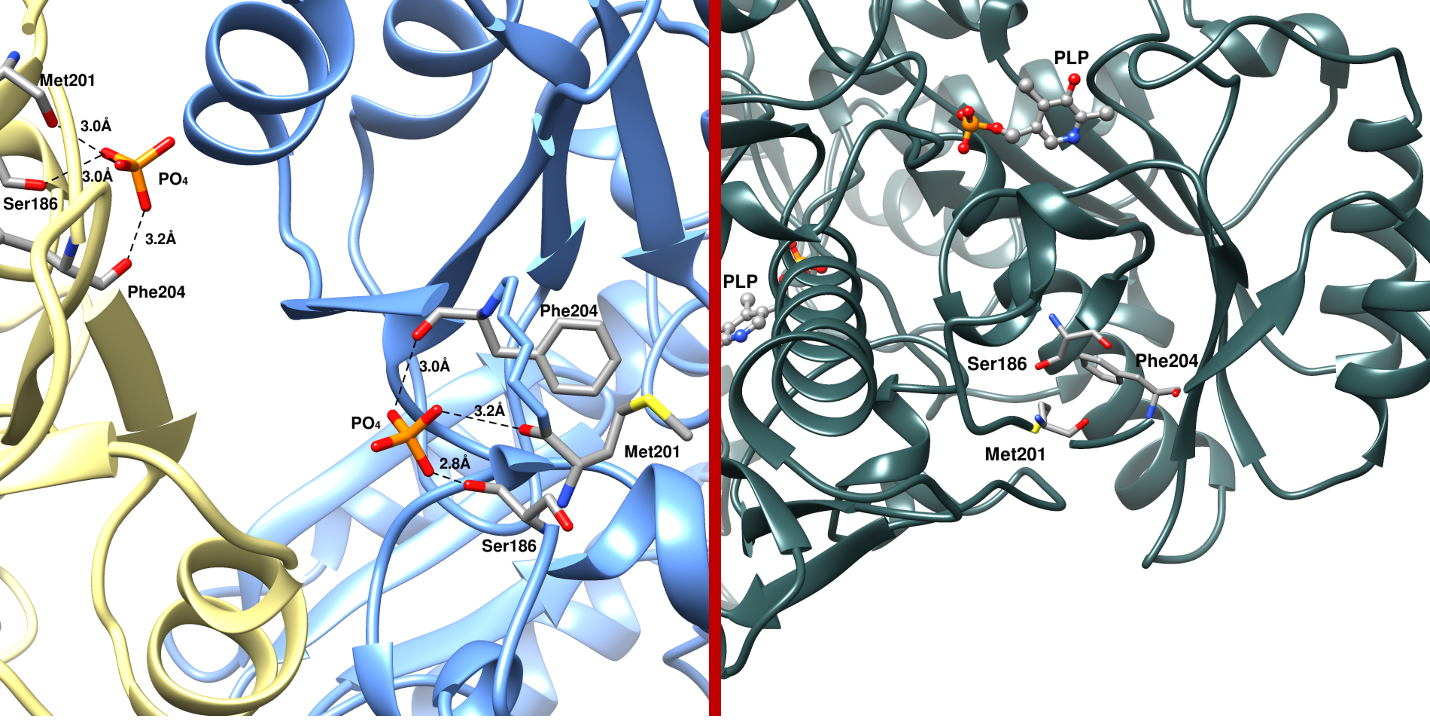


**Figure S14. Comparison of the phosphate binding site in crystal structure of *h*OAT soaked with AVA (left) and biological dimer of holoenzyme (right).**


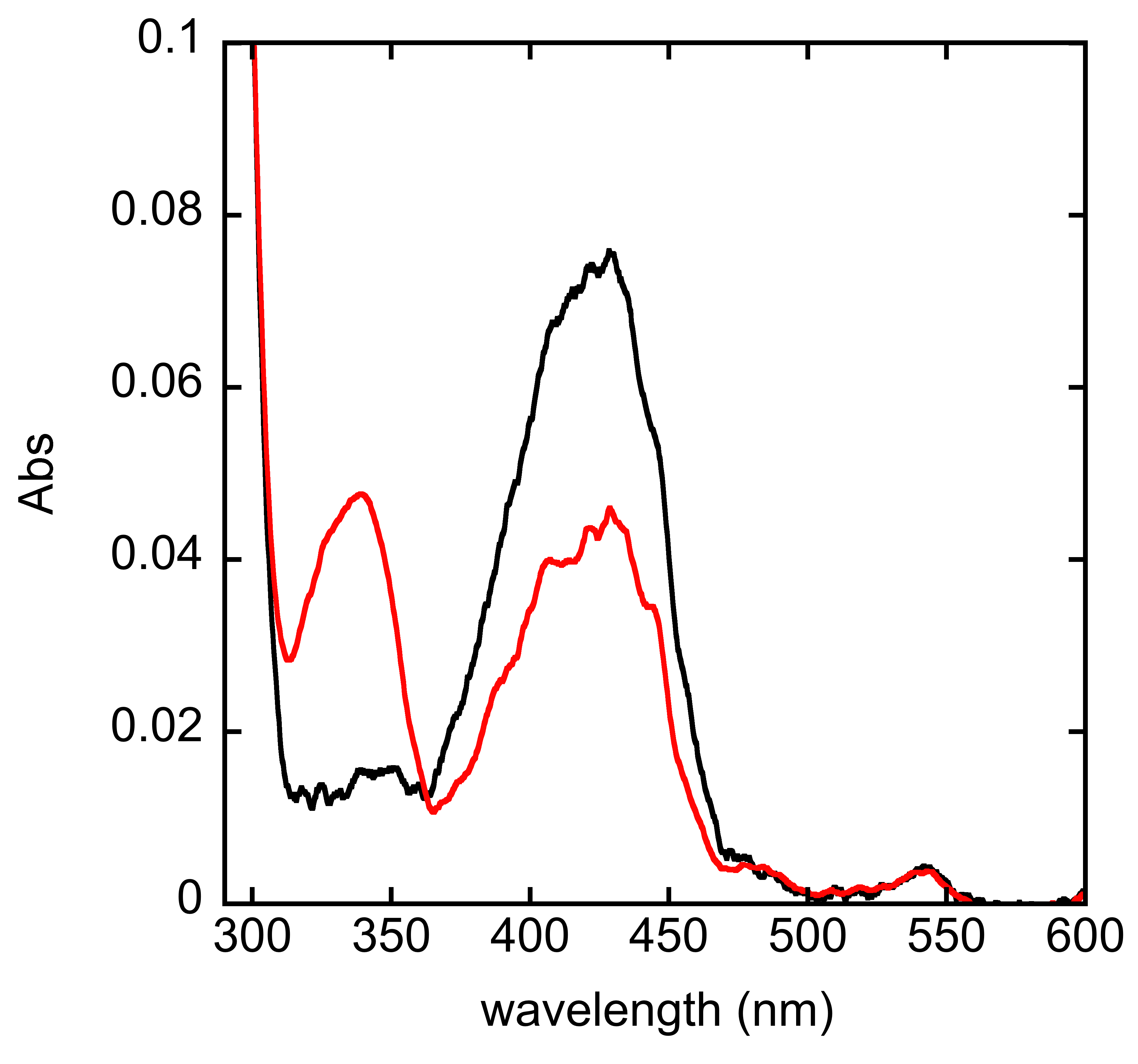


**Figure S15. Initial (black) and final (red) spectra of the reaction between 8 mM AVA and 9 µM *h*OAT.**
